# Mismatch screening in *Nicotiana benthamiana* to explore Pik‐1/Pik‐2 paired NLR platforms for receptor engineering

**DOI:** 10.1111/nph.70864

**Published:** 2025-12-28

**Authors:** Yuxuan Xi, Mark J. Banfield

**Affiliations:** ^1^ Department of Biochemistry and Metabolism John Innes Centre, Norwich Research Park Norwich NR4 7UH UK

**Keywords:** integrated domains, NLR immune receptors, pathogen effectors, plant immunity, protein engineering

## Disclaimer

The New Phytologist Foundation remains neutral with regard to jurisdictional claims in maps and in any institutional affiliations.

Nucleotide‐binding, leucine‐rich repeat (NLRs) proteins are the major class of plant intracellular immune receptors that recognise pathogen effectors, with activation triggering defence responses. Molecular engineering of NLRs can be an effective and reliable method to obtain new effector recognition specificities with the potential to tackle established and emerging plant diseases in modern agriculture (Zdrzalek *et al*., 2023; Dodds *et al*., 2024). Over the last decade, many NLRs carrying unconventional integrated domains (NLR‐IDs) have been reported (Marchal *et al*., 2022; Xi *et al*., 2022a; Shao *et al*., 2024). These IDs are targets for molecular engineering (De la Concepcion *et al*., 2018, 2019; Liu *et al*., 2021; Cesari *et al*., 2022; Bentham *et al*., 2023; Kourelis *et al*., 2023; Maidment *et al*., 2023; Zdrzalek *et al*., 2024; Zhang *et al*., 2024; Rim *et al*., 2025; H. Zhu *et al*., 2025; T. Zhu *et al*., 2025) as they directly mediate effector binding and are largely responsible for the ligand specificity that underpins receptor signalling (Cesari *et al*., 2013; Le Roux *et al*., 2015; Maqbool *et al*., 2015; Sarris *et al*., 2015; Ortiz *et al*., 2017; Zhang *et al*., 2017; De la Concepcion *et al*., 2018; Guo *et al*., 2018; Mukhi *et al*., 2021; Xi *et al*., 2022b).

One well‐studied plant NLR‐ID is the rice receptor Pik‐1 (Marchal *et al*., 2022; Xi *et al*., 2022a), which has an integrated heavy metal‐associated (HMA) domain between the coiled‐coil (CC) and nucleotide‐binding (NB) domains. The rice blast pathogen (*Magnaporthe oryzae*) effector AVR‐Pik is recognised by Pik‐1 through direct binding to Pik‐1^HMA^ (hence ‘sensor’ NLR), with the activation of immunity requiring the genetically linked paired ‘helper’ NLR, Pik‐2 (Ashikawa *et al*., 2008; Maqbool *et al*., 2015; Adachi *et al*., 2019; Zdrzalek *et al*., 2020). The rice NLR pair Pik and the rice blast effector AVR‐Pik both exist in allelic series in the host plant and pathogen population, respectively, having likely evolved from arms‐race co‐evolution since rice domestication (Kanzaki *et al*., 2012; Bialas *et al*., 2021). To date, at least 10 Pik alleles and 7 AVR‐Pik alleles have been identified and different Pik alleles encode different recognition specificities towards one or multiple AVR‐Pik variants (Qi *et al*., 2024).

Pik‐1 has been the focus of engineering studies to expand or otherwise alter the recognition specificities of Pik‐1 alleles. Structure‐guided mutagenesis of Pikp‐1^HMA^, including variants Pikp‐1^NK‐KE^ and Pikp‐1^SNK‐EKE^, expanded receptor recognition profiles to a wider range of AVR‐Pik variants, including those not previously recognised in nature (De la Concepcion *et al*., 2018, 2019; Maidment *et al*., 2023). Some alternative approaches applied high‐throughput random mutagenesis to Pikh‐1^HMA^ or Pikm‐1^HMA^ and obtained engineered Pikh‐1^HMA^ or Pikm‐1^HMA^ domains with new binding capabilities (Rim *et al*., 2025; H. Zhu *et al*., 2025). Another promising application is to swap the integrated Pik‐1^HMA^ domain with another protein, such as an effector host target. Three recent studies have swapped alternative HMA domains (OsHIPP19^HMA^, RGA5^HMA^ and OsHIPP43^HMA^) into the Pik‐1^HMA^ position, and these chimeric Pik‐1/Pik‐2 pairs enabled novel recognition specificities (Bentham *et al*., 2023; Maidment *et al*., 2023; Zdrzalek *et al*., 2024). Further, replacing the Pik^HMA^ domain with unrelated proteins, such as single‐chain nanobodies to GFP or mCherry, led to new receptors responsive to these proteins in plants (Kourelis *et al*., 2023). However, it is important that any Pik‐1^HMA^ modifications are evaluated in the context of the full‐length protein to ensure compatibility for function.

**Fig. 1:**
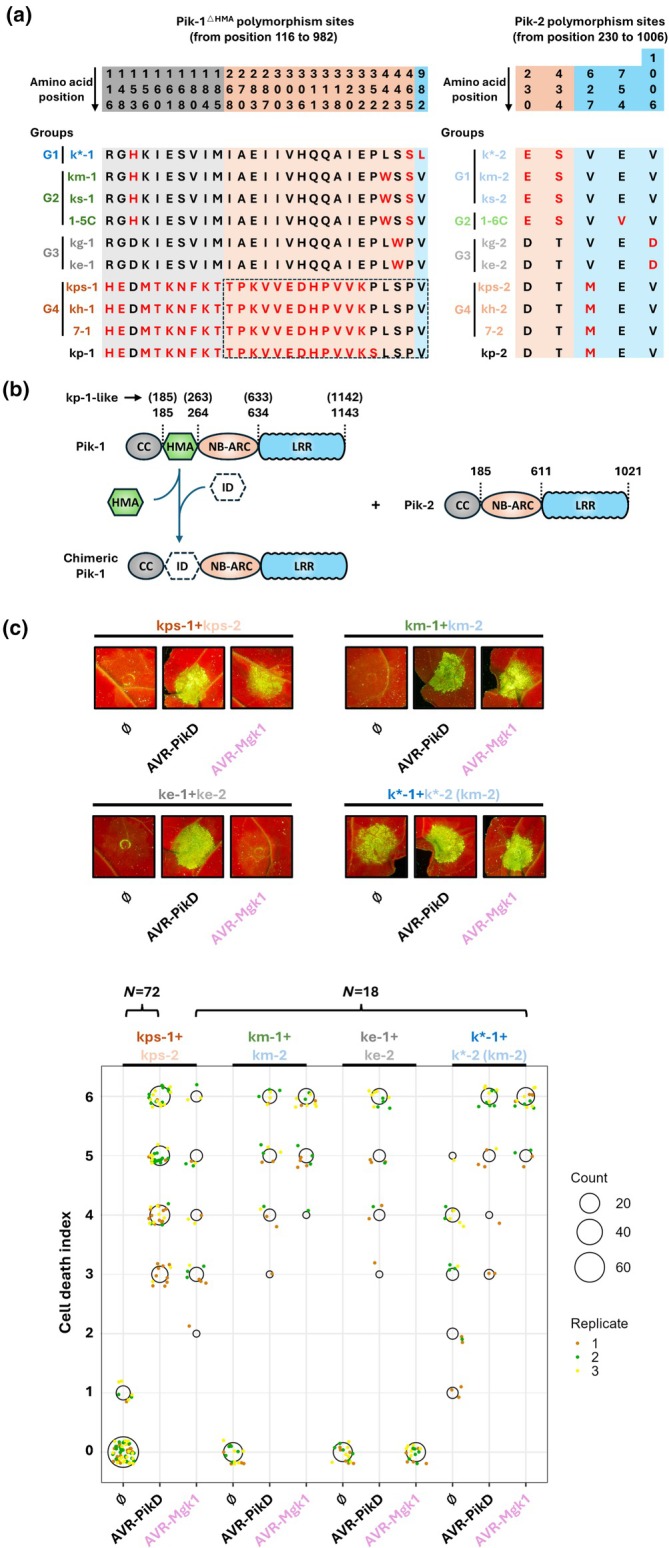
Pik pairs as resources for determining optimal ‘chassis’ for engineering recognition. (a) Polymorphisms of selected Pik‐1 (non‐heavy metal‐associated (HMA) region) and Pik‐2 variants. Both selected Pik‐1 and Pik‐2 variants are divided into four groups, and Pik‐1 and Pik‐2 variants from the same group are shown in the same colour. The upper part displays the positions of polymorphisms among 10 Pik‐1 (non‐HMA region) and Pik‐2 variants. The numbers are read vertically as indicated by the arrow. The lower part lists the amino acid polymorphisms. Red text highlights less common residues. Pikps‐1, Pikh‐1, Pi7‐1 and Pikp‐1 have a deletion at position 187 compared to the other six Pik‐1 variants. Therefore, the amino acid numbering in the dashed box is one less than the number marked above. For example, the first ‘T’ is position 267 of Pikp‐1‐like variants rather than 268. (b) Schematic diagrams of Pik‐1, chimeric Pik‐1 and Pik‐2. Wild‐type HMA domains can be replaced in the Pik‐1 chassis with other integrated domains (IDs) and co‐expressing chimeric Pik‐1 with compatible Pik‐2 may generate new effector specificities. The CC (coiled‐coil) domain, HMA (heavy metal‐associated) domain, NB‐ARC (nucleotide‐binding adaptor shared by Apaf1, certain R genes and CED4 family proteins), LRR (leucine‐rich repeat) domain and ID (integrated domain) are shown in different colours. (c) Selected allelic Pik pairs show expected effector recognition profiles in *Nicotiana benthamiana*. Phenotypes of four allelic Pik‐1/Pik‐2 pairs co‐expressed alone, or with either AVR‐PikD or AVR‐Mgk1, in *N. benthamiana* plants were monitored 5 d postinfiltration. The upper part shows representative leaf pictures taken under the UV light. The lower part presents the dot plot of cell death scores in each infiltrated area, ranging from 0 to 6 (De la Concepcion *et al*., 2018). Six technical replicates of each combination were performed in one experiment and experiments were repeated three times. The size of central circles for each score is proportional to the replicates' number and three biological replicates are distinguished by different colours.

Outside of direct Pik‐1 integrated domain engineering for effector recognition, a challenge for maintaining effective immune responses remains incompatibility between Pik‐1 chimeras/variants and Pik‐2. This can lead to effector‐independent responses in plants (often referred to as ‘autoactivation’) (Bialas *et al*., 2021; Bentham *et al*., 2023; Kourelis *et al*., 2023; Maidment *et al*., 2023; Zdrzalek *et al*., 2024). NLR autoactivity is an issue in engineered plants due to induced phenotypes such as stunted growth, dwarfism and necrosis in the absence of pathogen infection (Freh *et al*., 2022). For HMA domain insertions, one strategy to minimise the impact of autoactivation has been to modify the inserted domain while retaining effector‐dependent responses (Bialas *et al*., 2021; Maidment *et al*., 2023). However, this strategy is unlikely to be universally applicable to all potential integrated domains and is time‐consuming to overcome. Interestingly, we have demonstrated that a ‘mismatched’ Pikm‐1/Pikp‐2 pair, where the sensor NLR Pik‐1 was from the Pikm allelic variant and the helper NLR Pik‐2 was from the Pikp allelic variant, was more accommodating of alternative HMA domain integrations than the wild‐type Pikm‐1/Pikm‐2 allelic pair (De la Concepcion *et al*., 2019; Bentham *et al*., 2023; Zdrzalek *et al*., 2024). Most studies of Pik‐1 engineering have focussed on the Pikp and Pikm alleles. Given many other Pik alleles have been cloned that encode polymorphisms in Pik‐1 (outside the HMA domain) and Pik‐2 (Fig. 1a), we hypothesised that testing other mismatched pairings of Pik alleles could reveal an optimal ‘chassis’ for engineering.

To understand the potential of the mismatching strategy, we performed *Nicotiana benthamiana* cell death assays with multiple cloned Pik alleles (all Pik constructs were generated using the same promoter). Ultimately, an optimised engineering approach to introduce small proteins into the position of Pik‐1^HMA^ (Fig. 1b) would be agnostic to the integrated domain. Therefore, we treated wild‐type HMA domains as ‘insertions’ and their sequence differences were not considered in this screening. Based on the nine allelic Pik‐1/Pik‐2 pairs, we aligned sequences of Pik*‐1/Pik*‐2, Pikm‐1/Pikm‐2, Piks‐1/Piks‐2, Pi1‐5C/Pi1‐6C, Pikg‐1/Pikg‐2, Pike‐1/Pike‐2, Pikps‐1/Pikps‐2, Pikh‐1/Pikh‐2 and Pi7‐1/Pi7‐2 (Supporting Information Notes S1; Fig. 1a) and classified both Pik‐1 and Pik‐2 into four groups. We then selected one representative Pik‐1 (Pik*‐1, Pikm‐1, Pike‐1 and Pikps‐1) and Pik‐2 (Pikm‐2 (identical to Pik*‐2 and Piks‐2), Pi1‐6C, Pike‐2 (identical to Pikg‐2) and Pikps‐2 (identical to Pikh‐2 and Pi7‐2)) from each group for further study. The sequences of Pikp and Pikps variants differ by only one polymorphism in Pik‐1, while their Pik‐2 sequences are identical (Notes S1; Fig. 1a).

First, we investigated the recognition specificities of these four selected wild‐type Pik‐1 variants via monitoring immunity‐related signalling in *N. benthamiana*. As expected, co‐expression of the corresponding allelic Pik pairs with AVR‐PikD resulted in effector‐dependent cell death in all cases (Fig. 1c) (Kanzaki *et al*., 2012; Meng *et al*., 2021; Kovi *et al*., 2023). We also tested each pair for recognition of AVR‐Mgk1, a recently cloned MAX‐fold effector previously shown to be recognised by certain Pik variants (kp, km, k* and ks) (Sugihara *et al*., 2023; Xiao *et al*., 2023). Co‐expression of these Pik pairs with AVR‐Mgk1 resulted in effector‐dependent cell death in each case (Fig. 1c), except for Pike that showed no response. Expression of each NLR and the effectors was confirmed by western blot analyses, although accumulation of AVR‐Mgk1 was lower than AVR‐PikD (Fig. S1). As the amino acid sequence of the Pik‐1^HMA^ domain only differs by a single residue between Pikm‐1 and Pike‐1 (at position 229 a glutamine to aspartic acid change) (Notes S1), this highlights this site as important for the recognition of AVR‐Mgk1 in *N. benthamiana*.

During these experiments, we unexpectedly observed an autoactivation phenotype in the Pik* pair, in contrast with other Pik pairs (Fig. 1c). Such autoactivity with wild‐type Pik pairs has not previously been observed in *N. benthamiana*. The protein sequences of Pikm‐2 and Pik*‐2 are identical (Fig. 1a), and the main sequence differences between Pikm‐1 and Pik*‐1 are located at the C‐terminus of the HMA domain (Notes S1), where Pik*‐1^HMA^ carries a ‘GHAELLQ’ motif (position 251–257), which is only one amino acid difference compared to the sequence at the same position of the Pikp‐1^HMA^ (GDAELLQ, position 250–256). Interestingly, this region corresponds to the beginning of the *β*4 strand in the Pik‐1^HMA^ structure, a region recently shown to be involved in incompatibility between the Pikp and Pikm pairs by introducing *β*4 of Pikp‐1^HMA^ into the equivalent position of Pikm‐1^HMA^ (Bentham *et al*., 2023). We therefore hypothesised that the autoactivation phenotype of Pik* pair was likely linked to this HMA region although the sequences of Pikm‐1^*β*4^ and Pik*‐1^*β*4^ are not identical. To confirm that the various Pik‐1 and Pik‐2 variants do not independently trigger cell death, we expressed each of these receptors individually, confirming a lack of cell death with single proteins (Fig. S2).

**Fig. 2:**
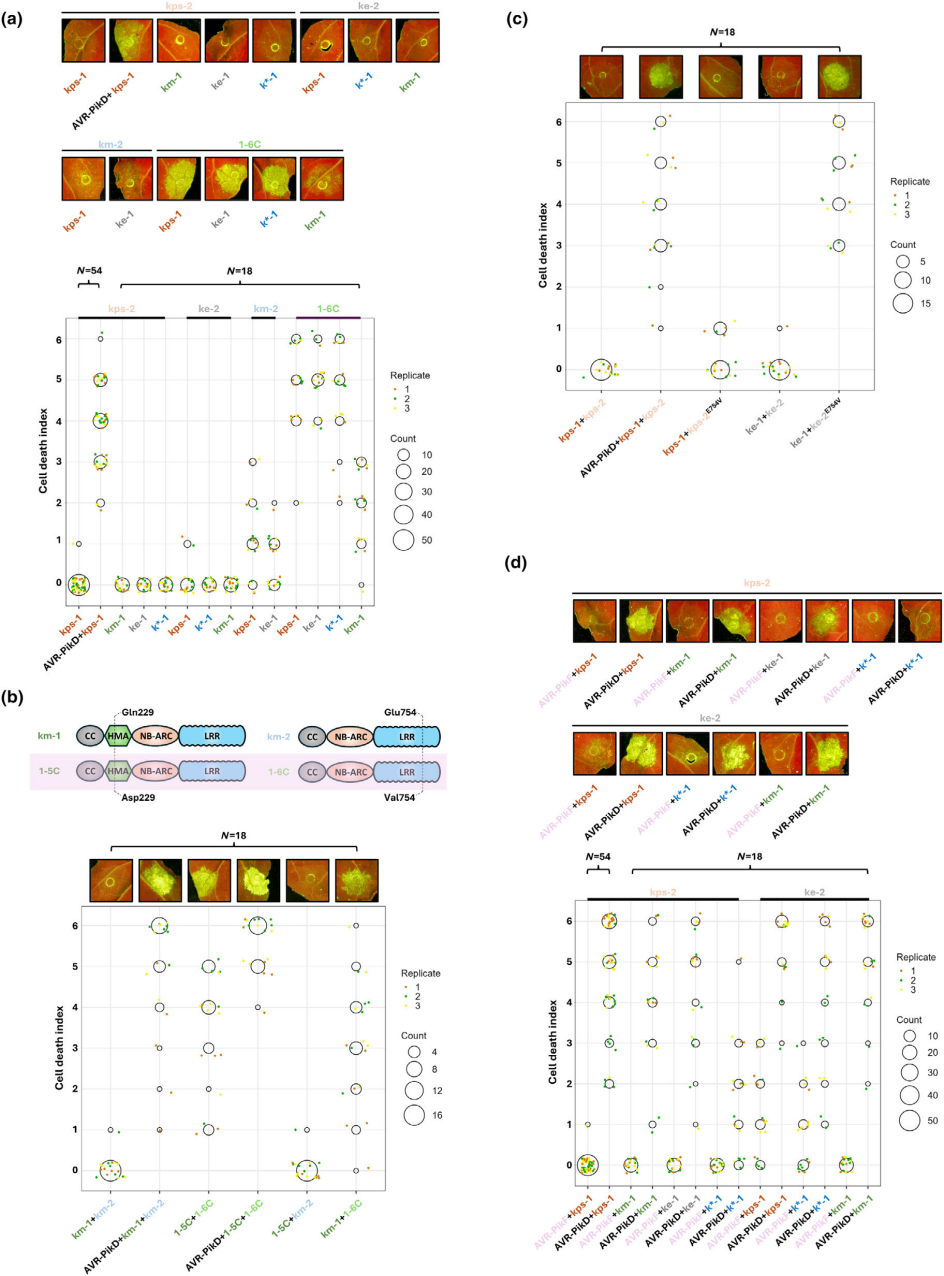
Mismatch screening in *Nicotiana benthamiana* to investigate Pik‐1/Pik‐2 paired Nucleotide‐binding, leucine‐rich repeat (NLR) chassis selection for engineering. (a) Autoactivation phenotypes for mismatched Pik‐1/Pik‐2 pairs reveal Pik‐2 dependencies. Pik‐1:HF variants co‐expressed with mismatched Pik‐2:HA variants in *N. benthamiana* with phenotypes recorded 5 d postinfiltration. (b) Val754 of Pi1‐6C underpins autoactivity of the Pi1‐5C/Pi1‐6C pair in *N. benthamiana*. Pi1‐5C and Pi1‐6C differ from Pikm‐1 and Pikm‐2, respectively, at one polymorphic site each. The performance of matching or mismatched pairs between Pikm and Pi1 alleles was tested in *N. benthamiana*. (c) Introduction of the E754V mutation into Pike‐2 induced autoactivation of the Pike pair in *N. benthamiana*. Results of co‐expression of Pikps‐1:HF/Pikps‐2^E754V^:HA and Pike‐1:HF/Pike‐2^E754V^:HA were monitored in *N. benthamiana* 5 d postinfiltration. (d) Mismatched Pik pairs including Pikps‐2 or Pike‐2 recognised AVR‐PikD in *N. benthamiana*. The mismatched Pik‐1:HF/Pikps‐2:HA or Pik‐1:HF/Pike‐2:HA pairs were co‐expressed with either AVR‐PikF or AVR‐PikD in *N. benthamiana* plants. For each figure, the upper part shows representative leaf pictures taken under UV light, and the lower part presents the dot plot of cell death scores in each infiltrated area, ranging from 0 to 6 (De la Concepcion *et al*., 2018). Six technical replicates of each combination were performed in one experiment and experiments were repeated three times. The size of central circles for each score is proportional to the replicates' number and three biological replicates are distinguished by different colours. CC, coiled‐coil; HMA, heavy metal‐associated; LRR, leucine‐rich repeat; NB‐ARC, nucleotide‐binding adaptor shared by Apaf1, certain R genes and CED4 family proteins.

After exploring the function of wild‐type Pik pairs, we co‐expressed each Pik‐1 with a nonallelic Pik‐2 to systematically examine their performance in *N. benthamiana* cell death assays (Fig. S3). We found that effector‐independent autoactivity was observed in a Pik‐2‐dependent manner, in which mismatched Pik pairs including Pikm‐2 or Pi1‐6C were autoactive, whereas those carrying Pikps‐2 or Pike‐2 were not (Fig. 2a). Sequence alignment of these four Pik‐2 variants revealed that both Pikps‐2 and Pike‐2 encode an aspartic acid and threonine at positions 230 and 434, respectively (Fig. 1a). These two amino acids were a focus of previous mutagenesis experiments investigating cell death responses of Pikp‐1^▵HMA^ in combination with reciprocal mutants (De la Concepcion *et al*., 2021; Bentham *et al*., 2023). In particular, the role of Pik‐2 Asp230 in suppressing autoactivity caused by incompatible Pik pairs was observed (De la Concepcion *et al*., 2021; Bentham *et al*., 2023). Therefore, we conclude that our new results showing Pik‐2‐dependent autoactivity are largely determined by this aspartic acid at position 230 in Pikps‐2 and Pike‐2.

We observed that Pikm‐2 or Pi1‐6C co‐expression with a mismatched Pik‐1 also showed different levels of effector‐independent autoactivity (Fig. 2a). Specifically, Pikps‐1 or Pike‐1 co‐expressed with Pikm‐2 displayed essentially weak cell death, while Pi1‐6C in combination with mismatched Pik‐1 showed strong cell death, although lower when co‐expressed with Pikm‐1. Pi1‐6C has a valine at position 754 (Fig. 1a), which is different to other Pik‐2 alleles that have a glutamic acid at this position. Pikm‐2 and Pi1‐6C only differ at this position, and as every other Pik‐1/Pi1‐6C pair elicited a stronger cell death response than induced by the same Pik‐1 in combination with Pikm‐2 (Figs 1c, 2a,b), we speculated that position 754 of Pik‐2 is important for regulating Pik‐1/Pik‐2 cell death. To test this hypothesis, we considered Pikm‐2 as a Pi1‐6C^V754E^ mutant and performed cell death assays in *N. benthamiana* to compare the performance of paired Pikm‐1/Pikm‐2, Pi1‐5C/Pi‐6C and mismatched Pikm‐1/Pi1‐6C, Pi1‐5C/Pikm‐2. As expected, Pi1‐5C/Pi1‐6C was autoactive and Pi1‐5C/Pikm‐2 did not show any responses, suggesting the importance of position 754 of Pik‐2 in Pik pair‐mediated cell death (Fig. 2b). Of note, the Pi1 pair was the second allelic Pik pair with an autoactivation phenotype we found in this study besides the Pik* pair, indicating allelic Pik pairs are not always compatible in *N. benthamiana*. Despite the autoactivity phenotype, the Pi1 pair is capable of AVR‐PikD recognition as we consistently observed elevated cell death on co‐expression of the Pi1 pair with the effector (Fig. 2b). We also introduced Asp230 into Pikm‐2 and Pi1‐6C and co‐expressed individual mutants with various Pik‐1 alleles (Fig. S4). Surprisingly, this single amino acid substitution is sufficient to abolish autoactivity when paired with the various Pik‐1 alleles (including the allelic Pik*‐1/Pik*‐2 (km‐2) and Pi1‐5C/Pi1‐6C pairs), further highlighting a critical regulatory role of Asp230 in autoactivation in Pik‐2. To further study the impact of the valine at position 754 in Pik‐2, we introduced a Glu754Val mutation into both Pikps‐2 and Pike‐2 and performed cell death assays of Pikps‐1/Pikps‐2^E754V^ and Pike‐1/Pike‐2^E754V^ in *N. benthamiana* (Fig. 2c). Pikps‐1/Pikps‐2^E754V^ displayed similar phenotypes to the wild‐type Pikps pair, but Pike‐1/Pike‐2^E754V^ triggered cell death, suggesting the residue at position 754 can be important for autoactivation, at least in the Pike pair. Therefore, retaining a glutamic acid at position 754 should limit autoactivation in an engineered Pik chassis.

We further selected mismatched Pik‐1/Pikps‐2 and Pik‐1/Pike‐2 pairs (that were previously shown to not display autoactivity phenotypes) for effector‐dependent cell death assays with both AVR‐PikD and AVR‐PikF (an AVR‐Pik variant not recognised by any cloned Pik alleles) (Fig. 2d). We observed that all the mismatched Pik‐1/Pikps‐2 pairs recognised AVR‐PikD but did not respond to AVR‐PikF. However, two out of three mismatched Pik‐1/Pike‐2 pairs, Pikps‐1/Pike‐2 and Pik*‐1/Pike‐2, weakly responded to AVR‐PikF. Moreover, Pik*‐1/Pikps‐2/AVR‐PikD only induced weak responses in plants, which is in line with expectations as Pik*‐1 carries a glutamic acid at position 229 that has been shown to reduce the responsiveness of Pik pair to AVR‐PikD (Sugihara *et al*., 2023).

Finally, we investigated the impact of replacing the wild‐type Pik‐1^HMA^ domain of four representative Pik‐1 variants with the RGA5^HMA^ domain on cell death phenotypes in *N. benthamiana* when paired with different Pik‐2 variants (except Pi1‐6C) (Fig. S5a,b). As previously observed (Bentham *et al*., 2023), the Pikm‐1^RGA5^/Pikm‐2 pair caused strong effector‐independent cell death, and we also observed cell death in the Pike background (Pike‐1^RGA5^/Pike‐2). Interestingly, this effector‐independent autoactivity was mitigated by mismatching with Pikps‐2 or Pikm‐2^E230D^. This shows that a mismatching strategy can be effective to overcome autoactivation resulting from noncognate integrated domain swapping.

In summary, we have systematically assessed the performance of mismatched representative Pik‐1 and Pik‐2 NLRs using the well‐established cell death assays in *N. benthamiana*. We found five mismatched Pik pairs (in addition to the Pikm‐1/Pikps‐2 (kp‐2) pair) that, although not paired with their natural partner, did not show autoactivity. All these six mismatched pairs contain either Pikps‐2 (kp‐2) or Pike‐2, both of which have an aspartic acid at position 230, a residue previously shown to be involved in the autoactivity of the Pik pair. Further, we introduced Asp230 into autoactive Pik pair combinations and observed a loss of this phenotype. This supports pairing engineered Pik‐1 with a Pik‐2 variant encoding Asp230 as a general strategy to restrict autoactivity. We confirmed that all six mismatched Pik pairs recognised the cognate effector AVR‐PikD. New to this work, we found a second site, position 754 in Pik‐2, that influences Pik‐1/Pik‐2 autoactivity. By testing the performance of five wild‐type allelic Pik pairs in *N. benthamiana* cell death assays, we unexpectedly revealed two combinations, the Pik* pair and Pi1 pair, that display autoactivity but still retain effector recognition (enhanced cell death in the presence of AVR‐PikD). How autoactivity is managed in rice cultivars encoding these two pairs is currently unknown. One explanation is their expression may be tightly regulated *in planta*, and are therefore not detrimental to growth. Alternatively, their activity may be regulated through posttranslational modifications, interaction with other proteins, or regulated protein turnover. In the future, resolving a structure of the Pik‐1/Pik‐2 pair will be essential to understand the roles of residues, such as those at positions 230 and 754 in Pik‐2, in receptor function. To this end, the allelic and mismatched Pik pairs tested in this study may serve as valuable targets for uncovering the structures of Pik pairs under different states. Such structures will undoubtedly suggest additional positions that could be targeted to fine‐tune Pik pair chassis to optimise their use in engineering disease resistance in crops.

## Materials and Methods

### Primers and constructs

Constructs for a subset of Pik pairs and effectors used in this study have been described previously (De la Concepcion *et al*., 2018; Maidment, 2020; Bentham *et al*., 2023) and are listed in Table S1. Pikps‐1 DOM2 acceptor was generated as described for Pikm‐1 DOM2 acceptor, except for the different gene background (Bentham *et al*., 2023). For newly assembled constructs, Level 0 vectors of the Pike pair, Pi1 pair, Pik*‐1 and Pik‐1^RGA5^ were generated by mutagenesis using LV0 constructs of Pikps‐2/Pikm‐2 or corresponding Pik‐1 DOM2 (Fig. S6) as templates, and primers and synthesised DNA fragments are listed in Tables S2 and S3, respectively. All constructs encoding Pik‐1 and Pik‐2 variants were generated with a C‐terminal HF (6×His/3×Flag) tag and a C‐terminal HA (6×HA) tag, respectively, and AVR effectors carry an N‐terminal Myc tag. The expression of NLR constructs was driven by Mas promoter/Mas terminator and the expression of effector constructs was driven by Ubiquitin‐10 promoter/35S terminator combination. All plasmid constructs were verified by DNA sequencing before use in experiments.

### *Nicotiana benthamiana* cell death assays

*Nicotiana benthamiana* plants were grown in a 22°C growth chamber under the 8 h : 16 h, dark : light cycle and 4‐ to 5‐wk‐old plants were used for cell death assays. LV1 constructs were transformed into *Agrobacterium tumefaciens* strain GV3101 and single colonies cultured in LB (Luria‐Bertani) media with carbenicillin/rifampicin/gentamicin resistance selection at 28°C. Following overnight growth, agrobacteria were collected by centrifugation and suspended in infiltration buffer (10 mM MgCl_2_, 10 mM MES (2‐(N‐morpholino)ethanesulfenic acid) (pH 5.6) supplemented with acetosyringone to a final concentration of 150 μM). NLRs, effectors and P19 were mixed at OD_600_ 0.4, 0.6 and 0.1, respectively, and total agrobacterial concentration was balanced by an empty vector transformant. Five days after infiltration, leaves were detached and imaged under UV light (abaxial side, for detailed camera setting see ‘Materials and methods’ in Bentham *et al*., 2023).

The cell death index of infiltrated areas was scored from 0 to 6 as shown in the supplemental document of De la Concepcion *et al*. (2018) and dot plots were generated using R v. 4.3.0 (https://www.r‐project.org) using the ggplot2 package (Wickham, 2016). All the cell death score data are shown in Table S4.

### Protein extraction and western blot

Agrobacteria carrying relevant constructs were diluted to OD_600_ 0.5 before infiltrating into leaves. Five leaf discs were harvested 2 d postinfiltration and frozen in liquid nitrogen. To extract proteins, frozen leaf discs were ground into powder resuspended in 200 μl extraction buffer (25 mM Tris–HCl (pH 7.5), 10% glycerol, 1 mM EDTA and 150 mM NaCl, 0.1% NP‐40 (Sigma), 10 mM DTT, 0.5% w/v PVPP and 1× protease inhibitor cocktail (Sigma)) (Zdrzalek *et al*., 2024). After clarification by 14 000 ***g*** centrifugation (30 min, 4°C), the supernatants were collected for SDS‐PAGE.

Following electrophoresis (4–20% Tris‐Glycine precast gels), proteins were transferred onto a PVDF (Polyvinylidene difluoride) membrane using a *Trans*‐Blot Turbo transfer system (Bio‐Rad). The membrane was then incubated with blocking buffer (5% w/v skimmed milk in TBS‐T (50 mM Tris–HCl pH 8.0, 150 mM NaCl, 0.1% Tween‐20)) for 1 h before incubating another 1 h with corresponding antibodies. For detecting three different tagged proteins, antibodies (primary HRP‐conjugated) were all diluted 3000‐fold in blocking buffer (α‐FLAG: Cohesion Biosciences; α‐HA: Invitrogen; α‐Myc: Santa Cruz Biotechnology). Subsequently, membranes were washed 3 × 10 min with TBS‐T buffer and signals detected using an ImageQuant LAS 500 spectrophotometer (GE Healthcare) (after adding Clarity Max Western ECL Substrate (Bio Rad) onto membranes). Ponceau staining was used to monitor the loading of total proteins.

## Competing interests

None declared.

## Author contributions

YX and MJB planned and designed the research; YX performed experiments; YX and MJB analysed the data and wrote the manuscript.

## Supporting information


**Fig. S1** Pik variants and effectors used in this study accumulate in *Nicotiana benthamiana*.
**Fig. S2** Pik‐1 or Pik‐2 proteins do not trigger cell death (autoactivation) in *Nicotiana benthamiana* when expressed separately.
**Fig. S3** Pairwise matrix for expression of selected Pik‐1 and Pik‐2 variants.
**Fig. S4** Introduction of the E230D mutation into Pikm‐2 (k*‐2) or Pi1‐6C can abolish autoactivation triggered by mismatched or allelic pairing between Pikm‐2 or Pi1‐6C and different Pik‐1 variants in *Nicotiana benthamiana*.
**Fig. S5** Autoactivation caused by Pik‐1^RGA5^ engineering can be reduced with a mismatching strategy.
**Fig. S6** Schematic diagram detailing generation of Pik‐1 and Pik‐2 variants by mutagenesis.
**Notes S1** Protein sequence alignments of Pik‐1 variants and Pik‐2 variants.
**Table S1** Constructs used in this study.
**Table S2** Primers used in this study.
**Table S3** Synthesised fragments used in this study.

**Table S4** Cell death scores of Figs 1(c), 2(a–d), S2, S4 and S5(a,b).
Please note: Wiley is not responsible for the content or functionality of any Supporting Information supplied by the authors. Any queries (other than missing material) should be directed to the *New Phytologist* Central Office.

## Data Availability

The data that support the findings of this study are available in the Supporting Information of this article. This includes data points used to prepare dot plots of Figs 1(c), 2a–d, S2, S4 and S5a,b (see Table S4).
