# Supplementary material for: Mismatch screening in Nicotiana benthamiana to explore Pik‐1/Pik‐2 paired NLR platforms for receptor engineering
Source: New Phytol. 2025 Dec 28;249(6):2623–9. doi: 10.1111/nph.70864 (PMC12917457; doi:10.1111/nph.70864)
Supplement: Supplementary file 1 — Fig. S1 Pik variants and effectors used in this study accumulate in Nicotiana benthamiana. Fig. S2 Pik‐1 or Pik‐2 proteins do not trigger cell death (autoactivation) in Nicotiana benthamiana when expressed separately. Fig. S3 Pairwise matrix for expression of selected Pik‐1 and Pik‐2 variants. Fig. S4 Introduction of the E230D mutation into Pikm‐2 (k*‐2) or Pi1‐6C can abolish autoactivation triggered by mismatched or allelic pairing between Pikm‐2 or Pi1‐6C and different Pik‐1 variants in Nicotiana benthamiana. Fig. S5 Autoactivation caused by Pik‐1RGA5 engineering can be reduced with a mismatching strategy. Fig. S6 Schematic diagram detailing generation of Pik‐1 and Pik‐2 variants by mutagenesis. Notes S1 Protein sequence alignments of Pik‐1 variants and Pik‐2 variants. Table S1 Constructs used in this study. Table S2 Primers used in this study. Table S3 Synthesised fragments used in this study. [file NPH-249-2623-s002.pdf]

New Phytologist Supporting Information

Article title: Mismatch screening in *Nicotiana benthamiana* to explore Pik-1/Pik-2 paired NLR platforms for receptor engineering

Authors: Yuxuan Xi & Mark J. Banfield

Article acceptance date: 05 December 2025

The following Supporting Information is available for this article:

Fig. S1 Pik variants and effectors used in this study accumulate in *N. benthamiana*.

Fig. S2 Pik-1 or Pik-2 proteins do not trigger cell death (autoactivation) in *N. benthamiana* when expressed separately.

Fig. S3 Pairwise matrix for expression of selected Pik-1 and Pik-2 variants.

Fig. S4 Introduction of the E230D mutation into Pikm-2 (k*-2) or Pi1-6C can abolish autoactivation triggered by mismatched or allelic pairing between Pikm-2 or Pi1-6C and different Pik-1 variants in *N. benthamiana*.

Fig. S5 Autoactivation caused by Pik-1^{RGA5} engineering can be reduced with a mismatching strategy.

Fig. S6 Schematic diagram detailing generation of Pik-1 and Pik-2 variants by mutagenesis.

Table S1 Constructs used in this study

Table S2 Primers used in this study

Table S3 Synthesised fragments used in this study

Table S4 Cell death scores of Fig. 1c, Fig. 2a-2d, Fig. S2, Fig. S4 and Fig. S5a-S5b

Notes S1 Protein sequence alignments of Pik-1 variants and Pik-2 variants

Fig. S1 Pik variants and effectors used in this study accumulate in *N. benthamiana*. All proteins were extracted from infiltrated leaf tissue 2 days post infiltration. Pik-1, Pik-2 or effectors were detected by western blot analyses using either anti-FLAG, anti-HA or anti-Myc antibodies, respectively. Ponceau staining indicates similar protein loading. The assays were repeated three times with similar results.

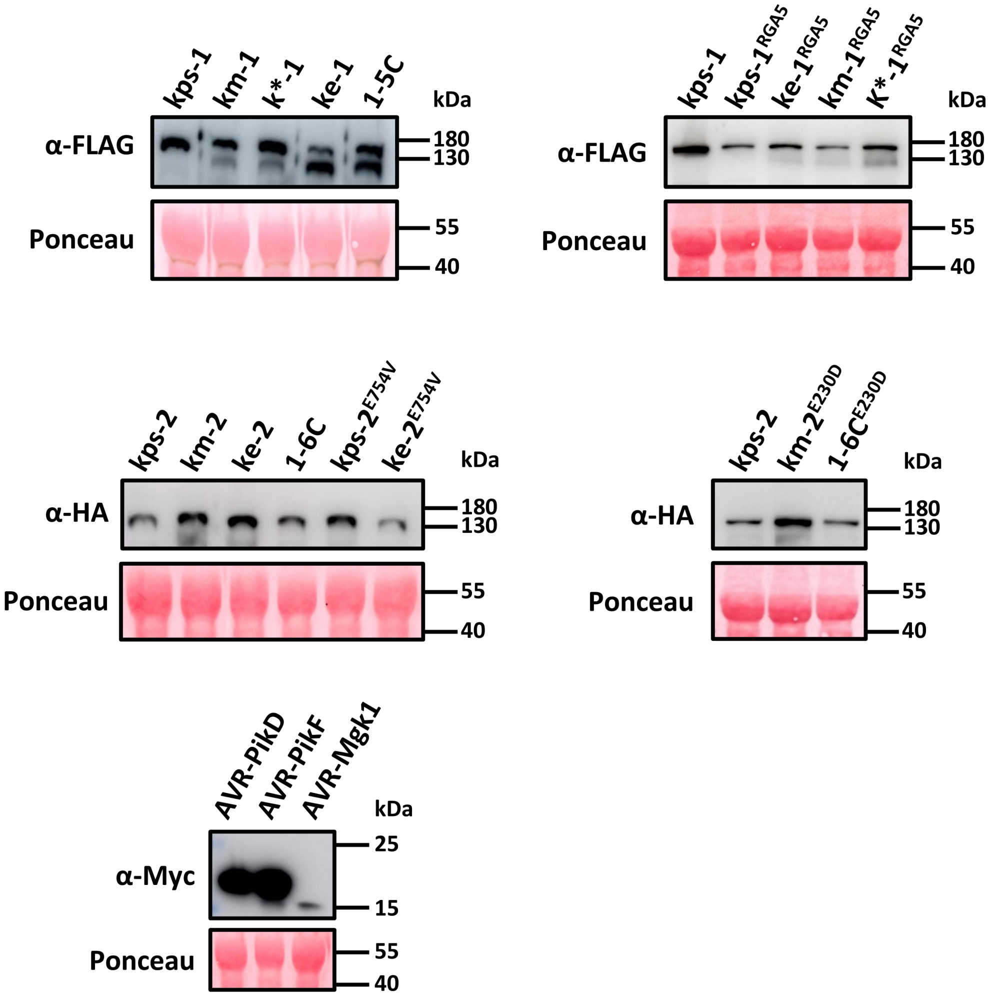

Fig. S2 Pik-1 or Pik-2 proteins do not trigger cell death (autoactivation) in *N. benthamiana* when expressed separately. Selected Pik-1 or Pik-2 variants were infiltrated into *N. benthamiana* leaves individually and phenotypes were monitored five days post infiltration. The upper part shows representative leaf pictures taken under the UV light. The lower part presents the dot plot of cell-death scores in each infiltrated area, ranging from 0-6 (De la Concepcion et al., 2018). Six technical replicates of each combination were performed in one experiment and experiments were repeated three times. The size of central circles for each score is proportional to the replicates' number and three biological replicates are distinguished by different colours.

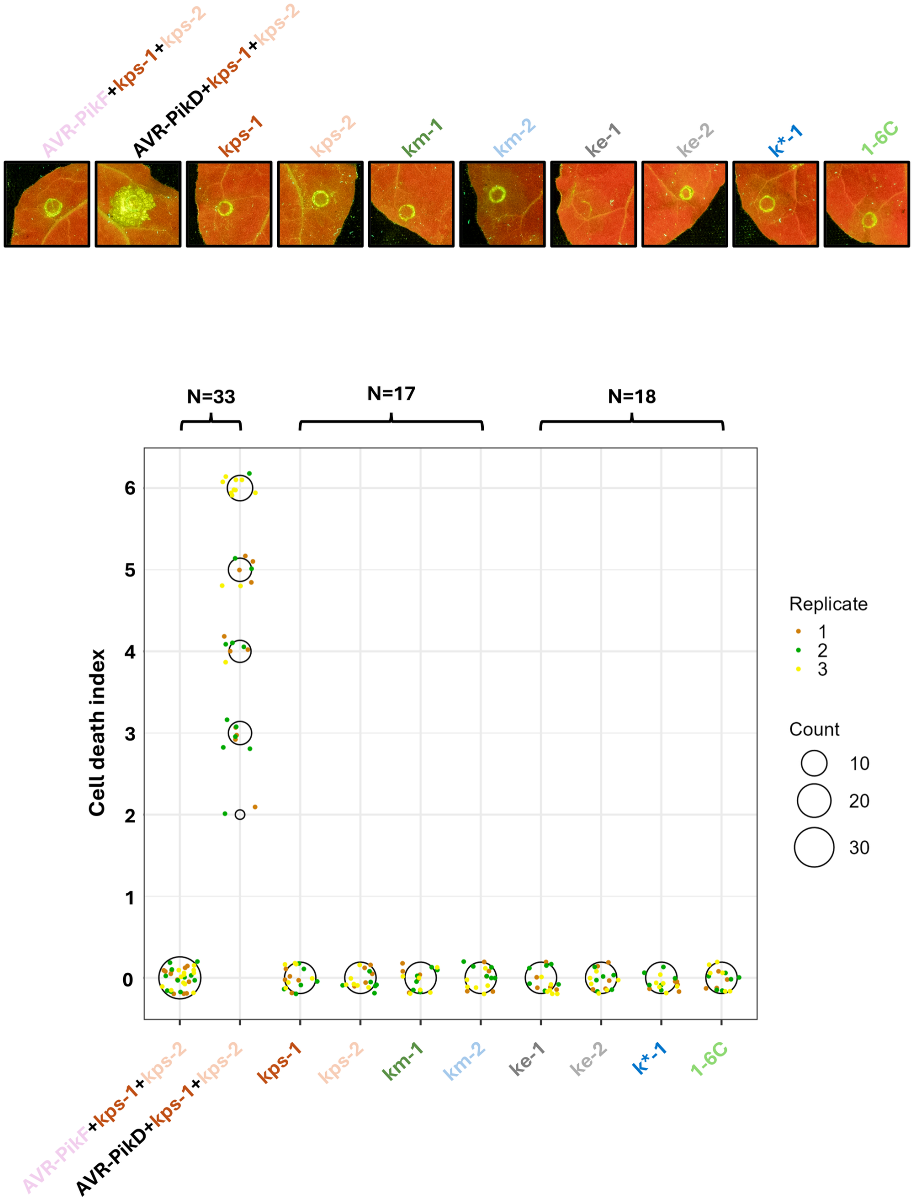

Fig. S3 Pairwise matrix for expression of selected Pik-1 and Pik-2 variants. "+" and "-" represent allelic Pik-1/Pik-2 pairs and mismatched pairs tested in this study, respectively.

Pik-1 \ Pik-2	km-2	1-6C	ke-2	kps-2
k*-1	+	-	-	-
km-1	+	-	-	-
ke-1	-	-	+	-
kps-1	-	-	-	+

+ Allelic pair

- Mismatched pair

Fig. S4 Introduction of the E230D mutation into Pikm-2 (k*-2) or Pi1-6C can abolish autoactivation triggered by mismatched or allelic pairing between Pikm-2 or Pi1-6C and different Pik-1 variants in *N. benthamiana*. Phenotypes were monitored five days post infiltration. The upper part shows representative leaf pictures taken under the UV light. The lower part presents the dot plot of cell-death scores in each infiltrated area, ranging from 0-6 (De la Concepcion et al., 2018). Six technical replicates of each combination were performed in one experiment and experiments were repeated three times. The size of central circles for each score is proportional to the replicates' number and three biological replicates are distinguished by different colours.

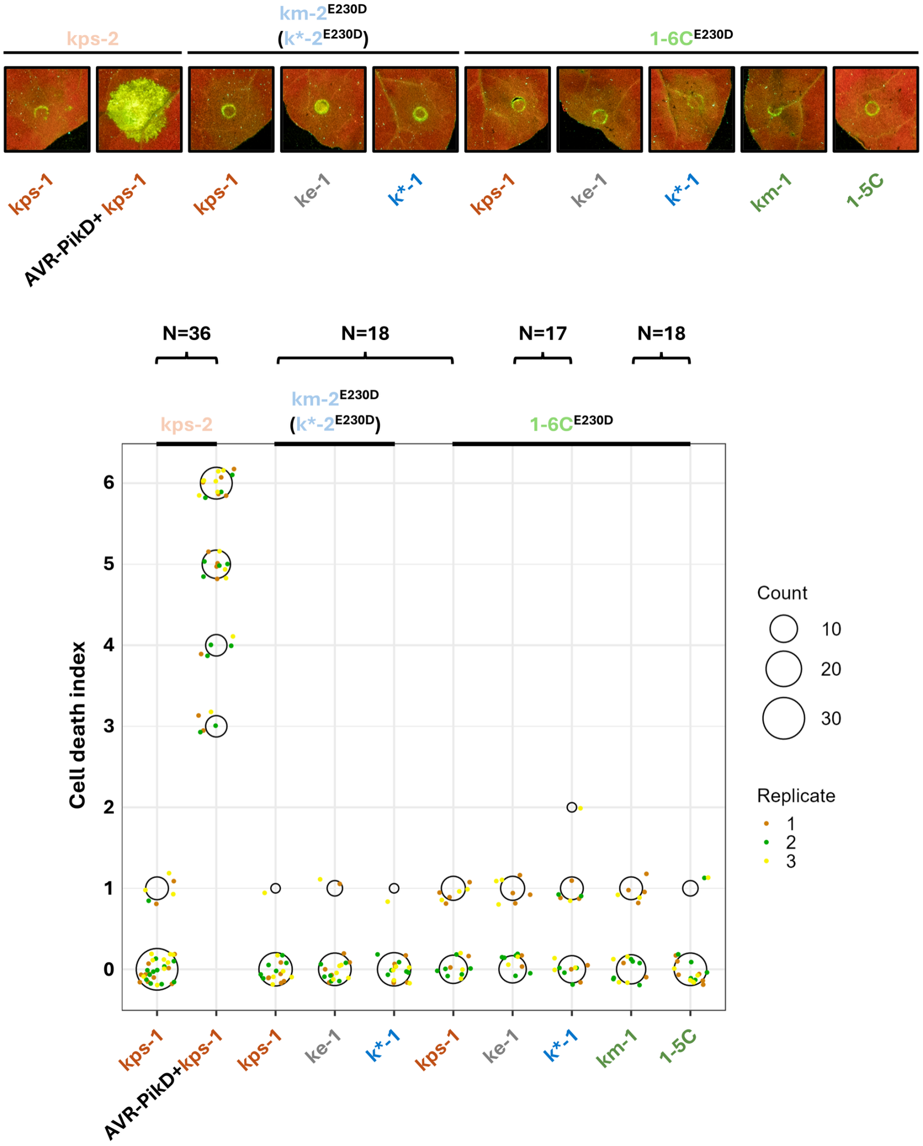

Fig. S5 Autoactivation caused by Pik-1^{RGA5} engineering can be reduced with a mismatching strategy. (a) Pikm-1^{RGA5}/Pikm-2 and Pike-1^{RGA5}/Pike-2 pairs triggered cell death in *N. benthamiana*. (b) Both autoactivation phenotypes of Pikm-1^{RGA5}/Pikm-2 and Pike-1^{RGA5}/Pike-2 were reduced by using either mismatched Pikps-2 or the Pikm-2^{E230D} mutant. Phenotypes were monitored five days post infiltration. The upper part shows representative leaf pictures taken under the UV light. The lower part presents the dot plot of cell-death scores in each infiltrated area, ranging from 0-6 (De la Concepcion et al., 2018). Six technical replicates of each combination were performed in one experiment and experiments were repeated three times. The size of central circles for each score is proportional to the replicates' number and three biological replicates are distinguished by different colours.

(a)

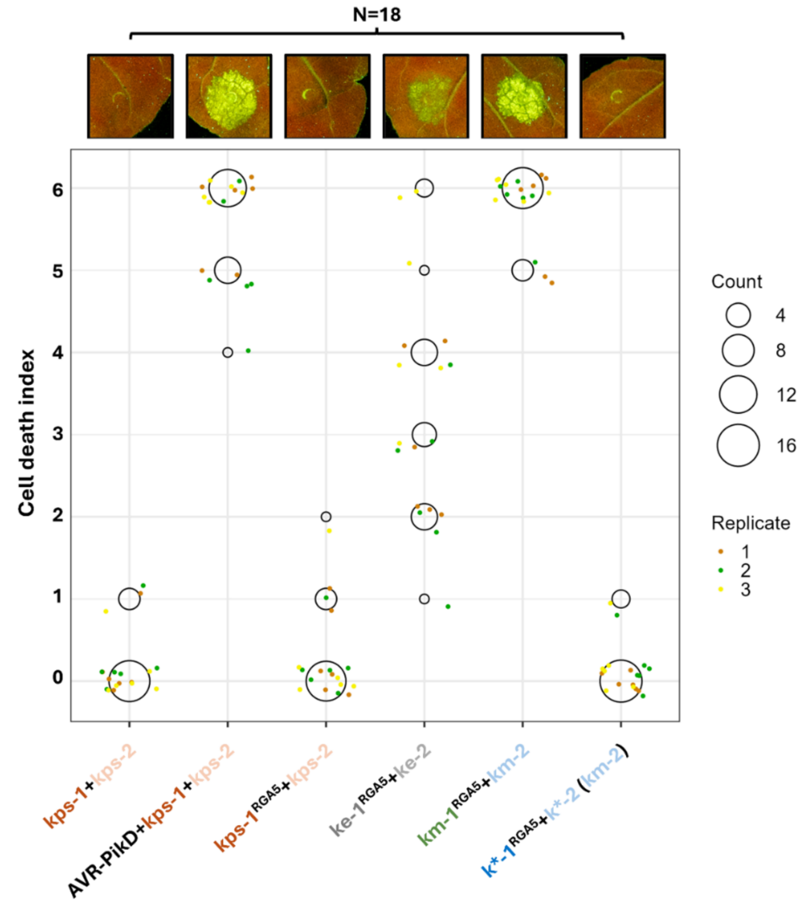

(b)

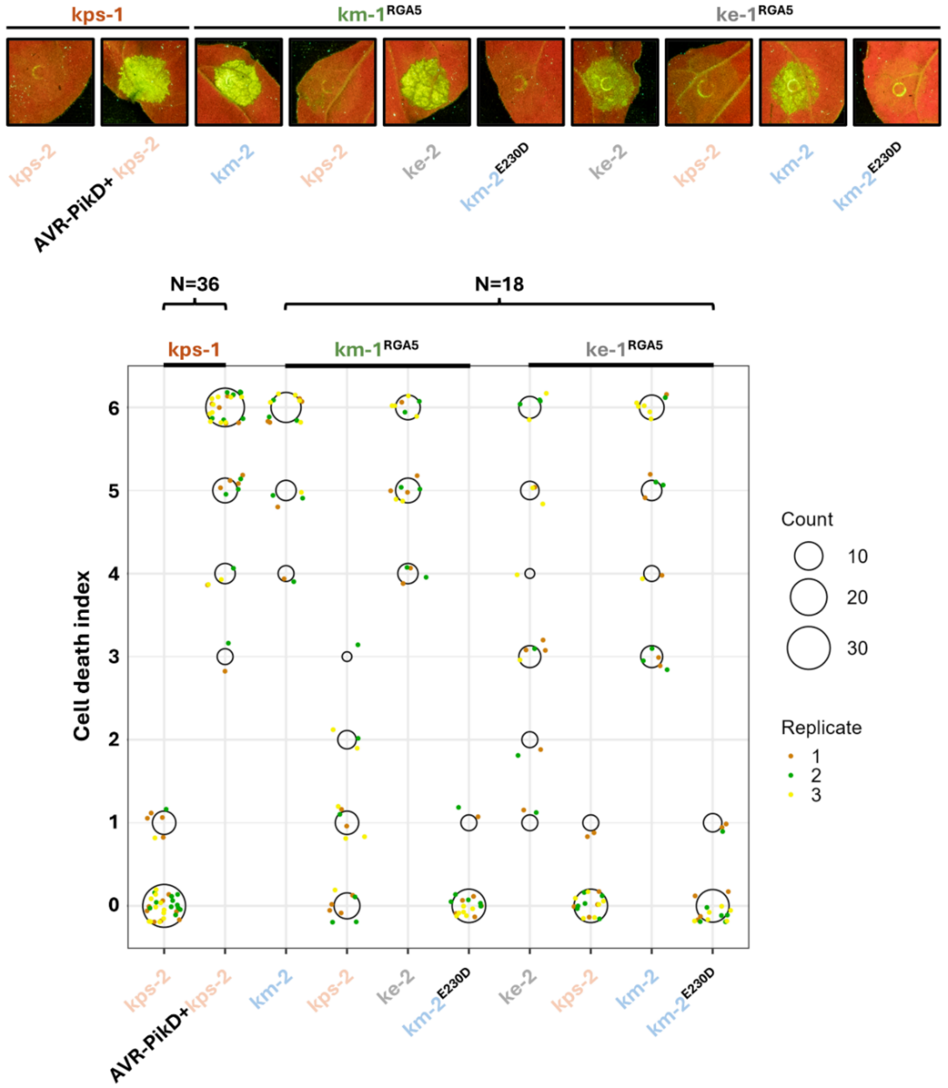
kps-1
km-1RGA5
ke-1RGA5
kps-2
AVR-PikD+ kps-2
km-2
kps-2
ke-2
km-2E230D
ke-2
kps-2
km-2
km-2E230D
N=36
N=18
kps-1
km-1RGA5
ke-1RGA5
Cell death index
6
5
4
3
2
1
0
Count
10
20
30
Replicate
1
2
3
kps-2
AVR-PikD+kps-2
km-2
kps-2
ke-2
km-2E230D
ke-2
kps-2
km-2
km-2E230D

Fig. S6 Schematic diagram detailing generation of Pik-1 and Pik-2 variants by mutagenesis. (a) Pik*-1 and Pike-1 were generated by multistep single-point mutagenesis of LV0 Pikm-1 DOM2 followed by insertion of the corresponding wild-type HMA domains using Golden Gate cloning. The wild-type $RGA5^{HMA}$ domain was fused into four LV0 Pik-1 DOM constructs to generate $Pikm\text{-}1^{RGA5}$, $Pikps\text{-}1^{RGA5}$, $Pik^*\text{-}1^{RGA5}$ and $Pike\text{-}1^{RGA5}$ using Golden Gate cloning. Pi1-5C was directly generated by one-step mutagenesis of Pikm-1 at position 229. (b) Pike-2 was derived from Pikps-2 by two-step mutagenesis, and other Val754- or Asp230-containing Pik-2 mutants were generated by one-step mutagenesis.

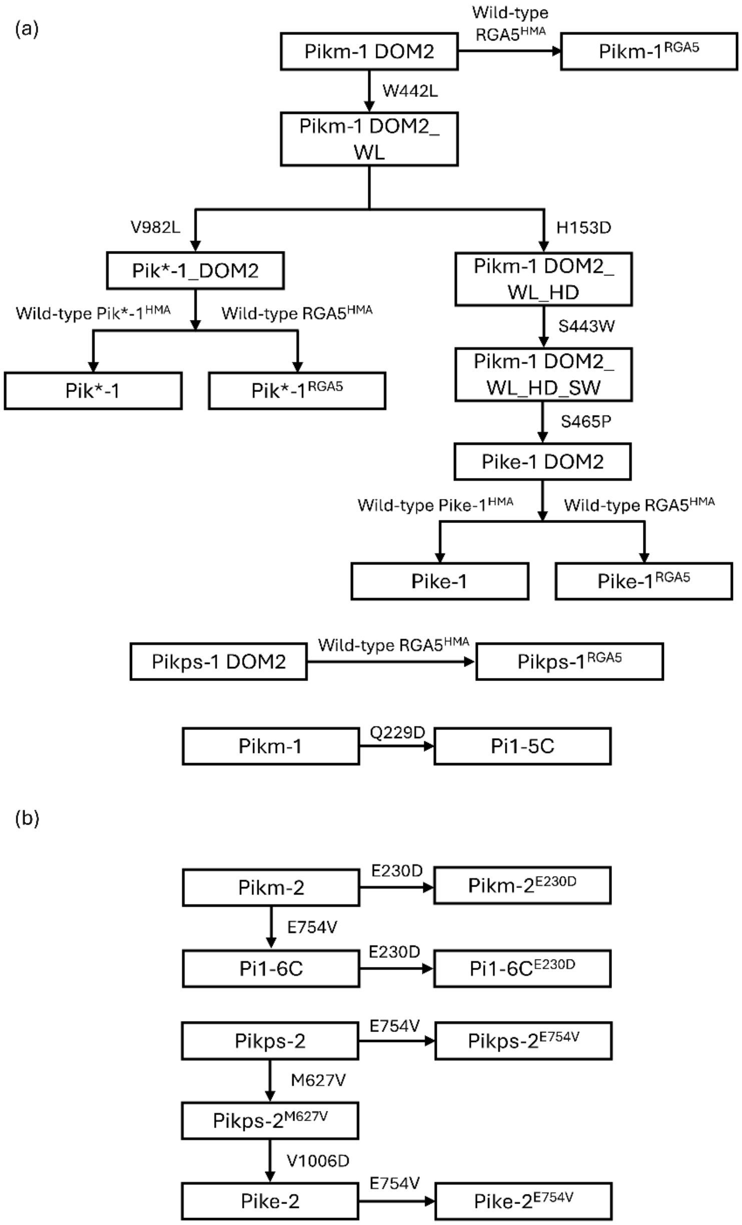

Table S1 Constructs used in this study

Name	Backbone	Resistance	Reference
Pikm-1 LV0	pICSL01005	Spectinomycin	De la Concepcion *et al.* (2018)
Pikps-1 LV0	pICSL01005	Spectinomycin	De la Concepcion *et al.* (2018)
Pikps-1 DOM2 LV0	pICSL01005	Spectinomycin	This study
Pikm-1 DOM2 LV0	pICSL01005	Spectinomycin	Bentham *et al.* (2023)
Pike-1 DOM2 LV0	pICSL01005	Spectinomycin	This study
Pik*-1 DOM2 LV0	pICSL01005	Spectinomycin	This study
Pike-1 LV0	pICSL01005	Spectinomycin	This study
Pik*-1 LV0	pICSL01005	Spectinomycin	This study
Pi-5C LV0	pICSL01005	Spectinomycin	This study
Pikps-1^{RGA5} LV0	pICSL01005	Spectinomycin	This study
Pikm-1^{RGA5} LV0	pICSL01005	Spectinomycin	Bentham *et al.* (2023)/ Recloned in this study for verification
Pike-1^{RGA5} LV0	pICSL01005	Spectinomycin	This study
Pik*-1^{RGA5} LV0	pICSL01005	Spectinomycin	This study
Pikm-2 (ks-2/k*-2) LV0	pICSL01005	Spectinomycin	De la Concepcion *et al.* (2018)
Pikps-2 (kp-2) LV0	pICSL01005	Spectinomycin	De la Concepcion *et al.* (2018)
Pike-2 LV0	pICSL01005	Spectinomycin	This study
Pi1-6C LV0	pICSL01005	Spectinomycin	This study
Pikps-2^{E754V} LV0	pICSL01005	Spectinomycin	This study
Pike-2^{E754V} LV0	pICSL01005	Spectinomycin	This study
Pikm-2^{E230D} LV0	pICSL01005	Spectinomycin	Bentham *et al.* (2023)/ Recloned in this study using Q5 mutagenesis kit
Pi1-6C^{E230D} LV0	pICSL01005	Spectinomycin	This study
Pikm-1:HF LV1	pICH47742	Carbenicillin	De la Concepcion *et al.* (2018)
Pikps-1:HF LV1	pICH47742	Carbenicillin	De la Concepcion *et al.* (2018)
Pik*-1:HF LV1	pICH47742	Carbenicillin	This study
Pike-1:HF LV1	pICH47742	Carbenicillin	This study
Pi1-5C:HF LV1	pICH47742	Carbenicillin	This study
Pikps-1^{RGA5}:HF LV1	pICH47742	Carbenicillin	This study
Pikm-1^{RGA5} :HF LV1	pICH47742	Carbenicillin	Bentham *et al.* (2023)/ Recloned in this study for verification
Pike-1^{RGA5}:HF LV1	pICH47742	Carbenicillin	This study
Pik*-1^{RGA5}:HF LV1	pICH47742	Carbenicillin	This study
Pikm-2 (ks-2/k*-2):HA	pICH47751	Carbenicillin	De la Concepcion *et al.* (2018)

LV1			
Pikps-2 (kp-2):HA LV1	pICH47751	Carbenicillin	De la Concepcion *et al.* (2018)
Pike-2:HA LV1	pICH47751	Carbenicillin	This study
Pi1-6C:HA LV1	pICH47751	Carbenicillin	This study
Pikps-2^{E754V} (kp-2^{E754V}):HA LV1	pICH47751	Carbenicillin	This study
Pike-2^{E754V}:HA LV1	pICH47751	Carbenicillin	This study
Pikm-2^{E230D}:HA LV1	pICH47751	Carbenicillin	Bentham *et al.* (2023)/ Recloned in this study for verification
Pi1-6C^{E230D}:HA LV1	pICH47751	Carbenicillin	This study
Myc:AVR-PikD LV1	pICH47751	Carbenicillin	De la Concepcion *et al.* (2018)
Myc:AVR-PikF LV1	Unknown LV1 backbone	Carbenicillin	Maidment. (2020) (p.180)
Myc:AVR-Mgk1 LV1	pICH47732	Carbenicillin	This study
Empty vector	pICH47742	Carbenicillin	Bentham *et al.* (2023)

Table S2 Primers used in this study

Name	Primer sequence	Method
2870_Pikm-1_cha_W442L_FWD	aatgatgctttgtcgttgtcttggg	Quick change Phusion amplification Ta=63℃
2871_Pikm-1_cha_W442L_REV	acgacaaagcatcattattatctagatccc	
2874_Pikm-1_cha_S443W_Pike1_FWD	atgatgctttgtggttgtcttggg	Quick change Phusion amplification Ta=63℃
2875_Pikm-1_cha_S443W_Pike1_REV	caaccacaaagcatcattattatctagatcc	
2876_Pikm-1_cha_S465P_Pike1_FWD	aatccatgctatgatattgtgaacatgtg	Quick change Phusion amplification Ta=63℃
2877_Pikm-1_cha_S465P_Pike1_REV	agcatggattatcctctccagttcc	
2878_Pikm-1_cha_H153D_Pike1_FWD	atggacaaacgtaaggaagagctcatc	Quick change Phusion amplification Ta=63℃
2879_Pikm-1_cha_H153D_Pike1_REV	tacgtttgtccatccccaccagc	
2898_Pikm-1_cha_V982L_Pik*1_FWD	CGACAGCCGGttgCCAAGGATAG	Q5 mutagenesis kit Ta=66.5℃
2900_Pikm-1_cha_V982L_Pik*1_REV	acacggaagctctccag	
2971_Pikm-1_Q229D_Pi1-5C_FWD	CCTAAGAGATgatGTTGTGGTGGTCG	Q5 mutagenesis kit Ta=63℃
2972_Pikm-1_Q229D_Pi1-5C_REV	TCACCGGCGATTGCA	
2913_Pik-2_E754V_FWD	CTTATCCCAAgtaAAAGGTAAAGG	Q5 mutagenesis kit Ta=56℃
2914_Pik-2_E754V_REV	AATCCTTTCTTTGCGG	
2859_Pikps2_M627V_FWD	ccatgtgccattccattcattcaac	Quick change Phusion amplification Ta=63℃
2860_Pikps2_M627V_REV	tggcacatggttcaggttcccaaac	
2861_Pikps2_V1006D_FWD	aaggataatactgagaaaccaaaag	Quick change Phusion amplification Ta=63℃
2862_Pikps2_V1006D_REV	tattatccttcaaatcatttacaag	
3233_Pik-2_E230D_FWD	AAAAGTCAGTgatAAATTTCAGTGC	Q5 mutagenesis kit Ta=58℃
3234_Pik-2_E230D_REV	CTGTACAATGCTGTGG	

Table S3 Synthesised fragments used in this study

Name	Sequence	Reference
Wild-type Pik*-1^{HMA}<br>(With overhangs)	AAGAAGACAACAGAataatgggaggggaaatgcaaaaaatcgtgttcaag<br>attcccatggtggacgataagagccgtacaaaagcaatgtcattggttgcaagtac<br>ggttggagtgcactcggttgcaatcgccggtgacctaagagacgaggttgtggtgg<br>tcggtgatggcattgactccatcaatctggtctctgcgctccggaagaaggtgggcc<br>atgcggagttgctgcaggtcagccaagtaaaggaaGATGAAGTCTTCAA	This study
Wild-type Pike-1^{HMA}<br>(With overhangs)	AAGAAGACAACAGAataatgggaggggaaatgcaaaaaatcgtgttcaag<br>attcccatggtggacgataagagccgtacaaaagcaatgtcattggttgcaagcac<br>ggttggagtgcactcggttgcaatcgccggtgacctaagagacgatgttgtggtggt<br>cggtgatggcattgactccatcaatctggtctctgcgctccggaagaaggtgggccc<br>tgcgatgtttctggaggtcagccaagtaaaggaaGATGAAGTCTTCAA	This study
Wild-type RGA5^{HMA}<br>(With overhangs)	AAAGAAGACAACAGAATAATGGGAGGGGAACGAACTAAGATA<br>GTTGTTAAGGTGCACATGCCATGCGGAAAATCCCGAGCAAAAG<br>CCATGGCGCTGGCTGCGTCAGTGAACGGGGTGGACAGCGTGG<br>AGATAACGGGGGAGGACAAAGACCGGCTGGTGGTGGTCGGCC<br>GTGGCATTGACCCTGTTCGCCTGGTGGCTCTCCTGCGCGAGAA<br>ATGTGGCCTCGCCGAGCTCTTGATGGTGGAGTTAGTTGAGAAA<br>GATGAAGTCTTCAAA	Bentham *et al.* (2023)

Notes S1 Protein sequence alignments of Pik-1 variants and Pik-2 variants

Pik-1 alignment

```
Pikh-1        MEAAAMAVTAATGALAPVLVKLAALLDDGECNLLEGSRSDAEFIRSELEAVHSLLTPNIL   60
Pikp-1        MEAAAMAVTAATGALAPVLVKLAALLDDGECNLLEGSRSDAEFIRSELEAVHSLLTPNIL   60
Pikps-1       MEAAAMAVTAATGALAPVLVKLAALLDDGECNLLEGSRSDAEFIRSELEAVHSLLTPNIL   60
Pi7-1         MEAAAMAVTAATGALAPVLVKLAALLDDGECNLLEGSRSDAEFIRSELEAVHSLLTPNIL   60
Pik*-1        MEAAAMAVTAATGALAPVLVKLAALLDDGECNLLEGSRSDAEFIRSELEAVHSLLTPNIL   60
Pike-1        MEAAAMAVTAATGALAPVLVKLAALLDDGECNLLEGSRSDAEFIRSELEAVHSLLTPNIL   60
Pikg-1        MEAAAMAVTAATGALAPVLVKLAALLDDGECNLLEGSRSDAEFIRSELEAVHSLLTPNIL   60
Pikm-1        MEAAAMAVTAATGALAPVLVKLAALLDDGECNLLEGSRSDAEFIRSELEAVHSLLTPNIL   60
Pi1-5C        MEAAAMAVTAATGALAPVLVKLAALLDDGECNLLEGSRSDAEFIRSELEAVHSLLTPNIL   60
Piks-1        MEAAAMAVTAATGALAPVLVKLAALLDDGECNLLEGSRSDAEFIRSELEAVHSLLTPNIL   60
              ************************************************************
                                            CC
Pikh-1        GRMGDDDAACKDGLIAEVRELSYDLDDAVDDFLELNFEQRRSASPFGELKARVEEHVSNR   120
Pikp-1        GRMGDDDAACKDGLIAEVRELSYDLDDAVDDFLELNFEQRRSASPFGELKARVEEHVSNR   120
Pikps-1       GRMGDDDAACKDGLIAEVRELSYDLDDAVDDFLELNFEQRRSASPFGELKARVEEHVSNR   120
Pi7-1         GRMGDDDAACKDGLIAEVRELSYDLDDAVDDFLELNFEQRRSASPFGELKARVEEHVSNR   120
Pik*-1        GRMGDDDAACKDGLIAEVRELSYDLDDAVDDFLELNFEQRRSASPFGELKARVEERVSNR   120
Pike-1        GRMGDDDAACKDGLIAEVRELSYDLDDAVDDFLELNFEQRRSASPFGELKARVEERVSNR   120
Pikg-1        GRMGDDDAACKDGLIAEVRELSYDLDDAVDDFLELNFEQRRSASPFGELKARVEERVSNR   120
Pikm-1        GRMGDDDAACKDGLIAEVRELSYDLDDAVDDFLELNFEQRRSASPFGELKARVEERVSNR   120
Pi1-5C        GRMGDDDAACKDGLIAEVRELSYDLDDAVDDFLELNFEQRRSASPFGELKARVEERVSNR   120
Piks-1        GRMGDDDAACKDGLIAEVRELSYDLDDAVDDFLELNFEQRRSASPFGELKARVEERVSNR   120
              *******************************************************:****
                                            CC
Pikh-1        FSDWKLPAASLPPSSVHRRAGLPPPDAELVGMDKRMEELTKLLEQGSNDASRWRKRKPHF   180
Pikp-1        FSDWKLPAASLPPSSVHRRAGLPPPDAELVGMDKRMEELTKLLEQGSNDASRWRKRKPHF   180
Pikps-1       FSDWKLPAASLPPSSVHRRAGLPPPDAELVGMDKRMEELTKLLEQGSNDASRWRKRKPHF   180
Pi7-1         FSDWKLPAASLPPSSVHRRAGLPPPDAELVGMDKRMEELTKLLEQGSNDASRWRKRKPHF   180
Pik*-1        FSDWKLPAASLPPSSVHRRAGLPPPDAGLVGMHKRKEELIELLEQGSSDASRWRKRKPHV   180
Pike-1        FSDWKLPAASLPPSSVHRRAGLPPPDAGLVGMDKRKEELIELLEQGSSDASRWRKRKPHV   180
Pikg-1        FSDWKLPAASLPPSSVHRRAGLPPPDAGLVGMDKRKEELIELLEQGSSDASRWRKRKPHV   180
Pikm-1        FSDWKLPAASLPPSSVHRRAGLPPPDAGLVGMHKRKEELIELLEQGSSDASRWRKRKPHV   180
Pi1-5C        FSDWKLPAASLPPSSVHRRAGLPPPDAGLVGMHKRKEELIELLEQGSSDASRWRKRKPHV   180
Piks-1        FSDWKLPAASLPPSSVHRRAGLPPPDAGLVGMHKRKEELIELLEQGSSDASRWRKRKPHV   180
              *************************** ****.** *** :******.***********.
                                            CC
Pikh-1        PLRKTG-LKQKIVIKVAMEGNNCRSKAMALVASTGGVDSVALVGDLRDKIEVVGYGIDPI   239
Pikp-1        PLRKTG-LKQKIVIKVAMEGNNCRSKAMALVASTGGVDSVALVGDLRDKIEVVGYGIDPI   239
Pikps-1       PLRKTG-LKQKIVIKVAMEGNNCRSKAMALVASTGGVDSVALVGDLRDKIEVVGYGIDPI   239
Pi7-1         PLRKTG-LKQKIVIKVAMEGNNCRSKAMALVASTGGVDSVALVGDLRDKIEVVGYGIDPI   239
Pik*-1        PLRIMGGEMQKIVFKIPMVDDKSRTKAMSLVASTVGVHSVAIAGDLRDEVVVVGDGIDSI   240
Pike-1        PLRIMGGEMQKIVFKIPMVDDKSRTKAMSLVASTVGVHSVAIAGDLRDDVVVVGDGIDSI   240
Pikg-1        PLRIMGGEMQKIVFKIPMVDDKSRTKAMSLVASTVGVHSVAIAGDLRDEVVVVGDGIDSI   240
Pikm-1        PLRIMGGEMQKIVFKIPMVDDKSRTKAMSLVASTVGVHSVAIAGDLRDQVVVVGDGIDSI   240
Pi1-5C        PLRIMGGEMQKIVFKIPMVDDKSRTKAMSLVASTVGVHSVAIAGDLRDDVVVVGDGIDSI   240
Piks-1        PLRIMGGEMQKIVFKIPMVDDKSRTKAMSLVASTVGVHSVAIAGDLRDEVVVVGDGIDSI   240
              ***  *   ****:*: * .::.*:***:***** **.***:.*****.: *** *** *
                185/185                            HMA
Pikh-1        KLISALRKKVGDAELLQVSQAKKDVKETTPMLAPVKSICEFHKVKTVCILGLPGGGKTTV   299
Pikp-1        KLISALRKKVGDAELLQVSQANKDVKETTPMLAPVKSICEFHKVKTVCILGLPGGGKTTV   299
Pikps-1       KLISALRKKVGDAELLQVSQANKDVKETTPMLAPVKSICEFHKVKTVCILGLPGGGKTTV   299
```

```
Pi7-1       KLISALRKKVGDAELLQVSQANKDVKETTPMLAPVKSICEFHKVKTVCILGLPGGGKTTV   299
Pik*-1      NLVSALRKKVGHAELLQVSQVKEDVKEITAMLAPVKSICEFHEVKTICILGLPGGGKTTI   300
Pike-1      NLVSALRKKVGPAMFLEVSQVKEDVKEITAMLAPVKSICEFHEVKTICILGLPGGGKTTI   300
Pikg-1      NLVSALRKKVGPAMFLEVSQVKEDVKEITAMLAPVKSICEFHEVKTICILGLPGGGKTTI   300
Pikm-1      NLVSALRKKVGPAMFLEVSQVKEDVKEITAMLAPVKSICEFHEVKTICILGLPGGGKTTI   300
Pi1-5C      NLVSALRKKVGPAMFLEVSQVKEDVKEITAMLAPVKSICEFHEVKTICILGLPGGGKTTI   300
Piks-1      NLVSALRKKVGPAMFLEVSQAKEDVKEITAMLAPVKSICEFHEVKTICILGLPGGGKTTI   300
            :*:******** * :*:***.::**** * ************:***:************:
                       HMA         263/264              NB-ARC
Pikh-1      ARELYDALGTHFPCRVFVSVSPSSSPSPNLTKTLADIFAQAQLGVTDTLSTPYGGSGTGR   359
Pikp-1      ARELYDALGTHFPCRVFVSVSPSSSPSPNLTKTLADIFAQAQLGVTDTLSTSYGGSGTGR   359
Pikps-1     ARELYDALGTHFPCRVFVSVSPSSSPSPNLTKTLADIFAQAQLGVTDTLSTPYGGSGTGR   359
Pi7-1       ARELYDALGTHFPCRVFVSVSPSSSPSPNLTKTLADIFAQAQLGVTDTLSTPYGGSGTGR   359
Pik*-1      ARVLYHALGTQFQCRVFASISPSSSPSPNLTETLADIFAQAQLGVTDTLSTPYGGSGTGR   360
Pike-1      ARVLYHALGTQFQCRVFASISPSSSPSPNLTETLADIFAQAQLGVTDTLSTPYGGSGTGR   360
Pikg-1      ARVLYHALGTQFQCRVFASISPSSSPSPNLTETLADIFAQAQLGVTDTLSTPYGGSGTGR   360
Pikm-1      ARVLYHALGTQFQCRVFASISPSSSPSPNLTETLADIFAQAQLGVTDTLSTPYGGSGTGR   360
Pi1-5C      ARVLYHALGTQFQCRVFASISPSSSPSPNLTETLADIFAQAQLGVTDTLSTPYGGSGTGR   360
Piks-1      ARVLYHALGTQFQCRVFASISPSSSPSPNLTETLADIFAQAQLGVTDTLSTPYGGSGTGR   360
            ** **.****:* ****.*:***********:******************* ********
                                         NB-ARC
Pikh-1      ALQQHLIDNISAFLLNKKYLIVIDDIWHWEEWEVIRKSIPKNDLGGRIIMTTRLNSIAEK   419
Pikp-1      ALQQHLIDNISAFLLNKKYLIVIDDIWHWEEWEVIRKSIPKNDLGGRIIMTTRLNSIAEK   419
Pikps-1     ALQQHLIDNISAFLLNKKYLIVIDDIWHWEEWEVIRKSIPKNDLGGRIIMTTRLNSIAEK   419
Pi7-1       ALQQHLIDNISAFLLNKKYLIVIDDIWHWEEWEVIRKSIPKNDLGGRIIMTTRLNSIAEK   419
Pik*-1      ALQQHLIDNISAFLLNKKYLIVIDDIWHWEEWEVIRKSIPKNDLGGRIIMTTRLNSIAEK   420
Pike-1      ALQQHLIDNISAFLLNKKYLIVIDDIWHWEEWEVIRKSIPKNDLGGRIIMTTRLNSIAEK   420
Pikg-1      ALQQHLIDNISAFLLNKKYLIVIDDIWHWEEWEVIRKSIPKNDLGGRIIMTTRLNSIAEK   420
Pikm-1      ALQQHLIDNISAFLLNKKYLIVIDDIWHWEEWEVIRKSIPKNDLGGRIIMTTRLNSIAEK   420
Pi1-5C      ALQQHLIDNISAFLLNKKYLIVIDDIWHWEEWEVIRKSIPKNDLGGRIIMTTRLNSIAEK   420
Piks-1      ALQQHLIDNISAFLLNKKYLIVIDDIWHWEEWEVIRKSIPKNDLGGRIIMTTRLNSIAEK   420
            ************************************************************
                                         NB-ARC
Pikh-1      CHTDDNDVFVYEVGDLDNNDALSLSWGIATKSGAGNRIGTGEDNPCYDIVNMCYGMPLAL   479
Pikp-1      CHTDDNDVFVYEVGDLDNNDALSLSWGIATKSGAGNRIGTGEDNPCYDIVNMCYGMPLAL   479
Pikps-1     CHTDDNDVFVYEVGDLDNNDALSLSWGIATKSGAGNRIGTGEDNPCYDIVNMCYGMPLAL   479
Pi7-1       CHTDDNDVFVYEVGDLDNNDALSLSWGIATKSGAGNRIGTGEDNPCYDIVNMCYGMPLAL   479
Pik*-1      CHTDDNDVFVYEVGDLDNNDALSLSWGIATKSGAGNRIGTGEDNSCYDIVNMCYGMPLAL   480
Pike-1      CHTDDNDVFVYEVGDLDNNDALWLSWGIATKSGAGNRIGTGEDNPCYDIVNMCYGMPLAL   480
Pikg-1      CHTDDNDVFVYEVGDLDNNDALWLSWGIATKSGAGNRIGTGEDNPCYDIVNMCYGMPLAL   480
Pikm-1      CHTDDNDVFVYEVGDLDNNDAWSLSWGIATKSGAGNRIGTGEDNSCYDIVNMCYGMPLAL   480
Pi1-5C      CHTDDNDVFVYEVGDLDNNDAWSLSWGIATKSGAGNRIGTGEDNSCYDIVNMCYGMPLAL   480
Piks-1      CHTDDNDVFVYEVGDLDNNDAWSLSWGIATKSGAGNRIGTGEDNSCYDIVNMCYGMPLAL   480
            *********************  ********************* ***************
                                         NB-ARC
Pikh-1      IWLSSALVGEIEELGGAEVKKCRDLRHIEDGILDIPSLQPLAESLCLGYNHLPLYLRTLL   539
Pikp-1      IWLSSALVGEIEELGGAEVKKCRDLRHIEDGILDIPSLQPLAESLCLGYNHLPLYLRTLL   539
Pikps-1     IWLSSALVGEIEELGGAEVKKCRDLRHIEDGILDIPSLQPLAESLCLGYNHLPLYLRTLL   539
Pi7-1       IWLSSALVGEIEELGGAEVKKCRDLRHIEDGILDIPSLQPLAESLCLGYNHLPLYLRTLL   539
Pik*-1      IWLSSALVGEIEELGGAEVKKCRDLRHIEDGILDIPSLQPLAESLCLGYNHLPLYLRTLL   540
Pike-1      IWLSSALVGEIEELGGAEVKKCRDLRHIEDGILDIPSLQPLAESLCLGYNHLPLYLRTLL   540
Pikg-1      IWLSSALVGEIEELGGAEVKKCRDLRHIEDGILDIPSLQPLAESLCLGYNHLPLYLRTLL   540
Pikm-1      IWLSSALVGEIEELGGAEVKKCRDLRHIEDGILDIPSLQPLAESLCLGYNHLPLYLRTLL   540
Pi1-5C      IWLSSALVGEIEELGGAEVKKCRDLRHIEDGILDIPSLQPLAESLCLGYNHLPLYLRTLL   540
Piks-1      IWLSSALVGEIEELGGAEVKKCRDLRHIEDGILDIPSLQPLAESLCLGYNHLPLYLRTLL   540
```

```
                ************************************************************
                                          NB-ARC
Pikh-1          LYCSAYHWSNRIERGRLVRRWIAEGFVSEEKEAEGYFGELINRGWITQHGDNNSYNYYEI   599
Pikp-1          LYCSAYHWSNRIERGRLVRRWIAEGFVSEEKEAEGYFGELINRGWITQHGDNNSYNYYEI   599
Pikps-1         LYCSAYHWSNRIERGRLVRRWIAEGFVSEEKEAEGYFGELINRGWITQHGDNNSYNYYEI   599
Pi7-1           LYCSAYHWSNRIERGRLVRRWIAEGFVSEEKEAEGYFGELINRGWITQHGDNNSYNYYEI   599
Pik*-1          LYCSAYHWSNRIERGRLVRRWIAEGFVSEEKEAEGYFGELINRGWITQHGDNNSYNYYEI   600
Pike-1          LYCSAYHWSNRIERGRLVRRWIAEGFVSEEKEAEGYFGELINRGWITQHGDNNSYNYYEI   600
Pikg-1          LYCSAYHWSNRIERGRLVRRWIAEGFVSEEKEAEGYFGELINRGWITQHGDNNSYNYYEI   600
Pikm-1          LYCSAYHWSNRIERGRLVRRWIAEGFVSEEKEAEGYFGELINRGWITQHGDNNSYNYYEI   600
Pi1-5C          LYCSAYHWSNRIERGRLVRRWIAEGFVSEEKEAEGYFGELINRGWITQHGDNNSYNYYEI   600
Piks-1          LYCSAYHWSNRIERGRLVRRWIAEGFVSEEKEAEGYFGELINRGWITQHGDNNSYNYYEI   600
                ************************************************************
                                          NB-ARC
Pikh-1          HPVMLAFLRCKSKEYNFLTCLGLGSDTSTSASSPRLIRRLSLQGGYPVDCLSSMSMDVSH   659
Pikp-1          HPVMLAFLRCKSKEYNFLTCLGLGSDTSTSASSPRLIRRLSLQGGYPVDCLSSMSMDVSH   659
Pikps-1         HPVMLAFLRCKSKEYNFLTCLGLGSDTSTSASSPRLIRRLSLQGGYPVDCLSSMSMDVSH   659
Pi7-1           HPVMLAFLRCKSKEYNFLTCLGLGSDTSTSASSPRLIRRLSLQGGYPVDCLSSMSMDVSH   659
Pik*-1          HPVMLAFLRCKSKEYNFLTCLGLGSDTSTSASSPRLIRRLSLQGGYPVDCLSSMSMDVSH   660
Pike-1          HPVMLAFLRCKSKEYNFLTCLGLGSDTSTSASSPRLIRRLSLQGGYPVDCLSSMSMDVSH   660
Pikg-1          HPVMLAFLRCKSKEYNFLTCLGLGSDTSTSASSPRLIRRLSLQGGYPVDCLSSMSMDVSH   660
Pikm-1          HPVMLAFLRCKSKEYNFLTCLGLGSDTSTSASSPRLIRRLSLQGGYPVDCLSSMSMDVSH   660
Pi1-5C          HPVMLAFLRCKSKEYNFLTCLGLGSDTSTSASSPRLIRRLSLQGGYPVDCLSSMSMDVSH   660
Piks-1          HPVMLAFLRCKSKEYNFLTCLGLGSDTSTSASSPRLIRRLSLQGGYPVDCLSSMSMDVSH   660
                ************************************************************
                            NB-ARC          633/634           LRR
Pikh-1          TCSLVVLGDVARPKGIPFYMFKRLRVLDLEDNKDIQDSHLQGICEQLSLRVRYLGLKGTR   719
Pikp-1          TCSLVVLGDVARPKGIPFYMFKRLRVLDLEDNKDIQDSHLQGICEQLSLRVRYLGLKGTR   719
Pikps-1         TCSLVVLGDVARPKGIPFYMFKRLRVLDLEDNKDIQDSHLQGICEQLSLRVRYLGLKGTR   719
Pi7-1           TCSLVVLGDVARPKGIPFYMFKRLRVLDLEDNKDIQDSHLQGICEQLSLRVRYLGLKGTR   719
Pik*-1          TCSLVVLGDVARPKGIPFYMFKRLRVLDLEDNKDIQDSHLQGICEQLSLRVRYLGLKGTR   720
Pike-1          TCSLVVLGDVARPKGIPFYMFKRLRVLDLEDNKDIQDSHLQGICEQLSLRVRYLGLKGTR   720
Pikg-1          TCSLVVLGDVARPKGIPFYMFKRLRVLDLEDNKDIQDSHLQGICEQLSLRVRYLGLKGTR   720
Pikm-1          TCSLVVLGDVARPKGIPFYMFKRLRVLDLEDNKDIQDSHLQGICEQLSLRVRYLGLKGTR   720
Pi1-5C          TCSLVVLGDVARPKGIPFYMFKRLRVLDLEDNKDIQDSHLQGICEQLSLRVRYLGLKGTR   720
Piks-1          TCSLVVLGDVARPKGIPFYMFKRLRVLDLEDNKDIQDSHLQGICEQLSLRVRYLGLKGTR   720
                ************************************************************
                                           LRR
Pikh-1          IRKLPQEMRKLKHLEILYVGSTRISELPQEIGELKHLRILDVRNTDITELPLQIRELQHL   779
Pikp-1          IRKLPQEMRKLKHLEILYVGSTRISELPQEIGELKHLRILDVRNTDITELPLQIRELQHL   779
Pikps-1         IRKLPQEMRKLKHLEILYVGSTRISELPQEIGELKHLRILDVRNTDITELPLQIRELQHL   779
Pi7-1           IRKLPQEMRKLKHLEILYVGSTRISELPQEIGELKHLRILDVRNTDITELPLQIRELQHL   779
Pik*-1          IRKLPQEMRKLKHLEILYVGSTRISELPQEIGELKHLRILDVRNTDITELPLQIRELQHL   780
Pike-1          IRKLPQEMRKLKHLEILYVGSTRISELPQEIGELKHLRILDVRNTDITELPLQIRELQHL   780
Pikg-1          IRKLPQEMRKLKHLEILYVGSTRISELPQEIGELKHLRILDVRNTDITELPLQIRELQHL   780
Pikm-1          IRKLPQEMRKLKHLEILYVGSTRISELPQEIGELKHLRILDVRNTDITELPLQIRELQHL   780
Pi1-5C          IRKLPQEMRKLKHLEILYVGSTRISELPQEIGELKHLRILDVRNTDITELPLQIRELQHL   780
Piks-1          IRKLPQEMRKLKHLEILYVGSTRISELPQEIGELKHLRILDVRNTDITELPLQIRELQHL   780
                ************************************************************
                                           LRR
Pikh-1          HTLDVRNTPISELPPQVGKLQNLKIMCVRSTGVRELPKEIGELNHLQTLDVRNTRVRELP   839
Pikp-1          HTLDVRNTPISELPPQVGKLQNLKIMCVRSTGVRELPKEIGELNHLQTLDVRNTRVRELP   839
Pikps-1         HTLDVRNTPISELPPQVGKLQNLKIMCVRSTGVRELPKEIGELNHLQTLDVRNTRVRELP   839
Pi7-1           HTLDVRNTPISELPPQVGKLQNLKIMCVRSTGVRELPKEIGELNHLQTLDVRNTRVRELP   839
Pik*-1          HTLDVRNTPISELPPQVGKLQNLKIMCVRSTGVRELPKEIGELNHLQTLDVRNTRVRELP   840
Pike-1          HTLDVRNTPISELPPQVGKLQNLKIMCVRSTGVRELPKEIGELNHLQTLDVRNTRVRELP   840
```

```
Pikg-1      HTLDVRNTPISELPPQVGKLQNLKIMCVRSTGVRELPKEIGELNHLQTLDVRNTRVRELP   840
Pikm-1      HTLDVRNTPISELPPQVGKLQNLKIMCVRSTGVRELPKEIGELNHLQTLDVRNTRVRELP   840
Pi1-5C      HTLDVRNTPISELPPQVGKLQNLKIMCVRSTGVRELPKEIGELNHLQTLDVRNTRVRELP   840
Piks-1      HTLDVRNTPISELPPQVGKLQNLKIMCVRSTGVRELPKEIGELNHLQTLDVRNTRVRELP   840
            ************************************************************
                                        LRR
Pikh-1      WQAGQISQSLRVLAGDSGDGVRLPEGVCEALINGIPGATRAKCREVLSIAIIDRFGPPLV   899
Pikp-1      WQAGQISQSLRVLAGDSGDGVRLPEGVCEALINGIPGATRAKCREVLSIAIIDRFGPPLV   899
Pikps-1     WQAGQISQSLRVLAGDSGDGVRLPEGVCEALINGIPGATRAKCREVLSIAIIDRFGPPLV   899
Pi7-1       WQAGQISQSLRVLAGDSGDGVRLPEGVCEALINGIPGATRAKCREVLSIAIIDRFGPPLV   899
Pik*-1      WQAGQISQSLRVLAGDSGDGVRLPEGVCEALINGIPGATRAKCREVLSIAIIDRFGPPLV   900
Pike-1      WQAGQISQSLRVLAGDSGDGVRLPEGVCEALINGIPGATRAKCREVLSIAIIDRFGPPLV   900
Pikg-1      WQAGQISQSLRVLAGDSGDGVRLPEGVCEALINGIPGATRAKCREVLSIAIIDRFGPPLV   900
Pikm-1      WQAGQISQSLRVLAGDSGDGVRLPEGVCEALINGIPGATRAKCREVLSIAIIDRFGPPLV   900
Pi1-5C      WQAGQISQSLRVLAGDSGDGVRLPEGVCEALINGIPGATRAKCREVLSIAIIDRFGPPLV   900
Piks-1      WQAGQISQSLRVLAGDSGDGVRLPEGVCEALINGIPGATRAKCREVLSIAIIDRFGPPLV   900
            ************************************************************
                                        LRR
Pikh-1      GIFKVPGSHMRIPKMIKDHFRVLSCLDIRLCHKLEDDDQKFLAEMPNLQTLVLRFEALPR   959
Pikp-1      GIFKVPGSHMRIPKMIKDHFRVLSCLDIRLCHKLEDDDQKFLAEMPNLQTLVLRFEALPR   959
Pikps-1     GIFKVPGSHMRIPKMIKDHFRVLSCLDIRLCHKLEDDDQKFLAEMPNLQTLVLRFEALPR   959
Pi7-1       GIFKVPGSHMRIPKMIKDHFRVLSCLDIRLCHKLEDDDQKFLAEMPNLQTLVLRFEALPR   959
Pik*-1      GIFKVPGSHMRIPKMIKDHFRVLSCLDIRLCHKLEDDDQKFLAEMPNLQTLVLRFEALPR   960
Pike-1      GIFKVPGSHMRIPKMIKDHFRVLSCLDIRLCHKLEDDDQKFLAEMPNLQTLVLRFEALPR   960
Pikg-1      GIFKVPGSHMRIPKMIKDHFRVLSCLDIRLCHKLEDDDQKFLAEMPNLQTLVLRFEALPR   960
Pikm-1      GIFKVPGSHMRIPKMIKDHFRVLSCLDIRLCHKLEDDDQKFLAEMPNLQTLVLRFEALPR   960
Pi1-5C      GIFKVPGSHMRIPKMIKDHFRVLSCLDIRLCHKLEDDDQKFLAEMPNLQTLVLRFEALPR   960
Piks-1      GIFKVPGSHMRIPKMIKDHFRVLSCLDIRLCHKLEDDDQKFLAEMPNLQTLVLRFEALPR   960
            ************************************************************
                                        LRR
Pikh-1      QPITINGTGFQMLESFRVDSRVPRIAFHEDAMPNLKLLEFKFYAGPASNDAIGITNLKSL   1019
Pikp-1      QPITINGTGFQMLESFRVDSRVPRIAFHEDAMPNLKLLEFKFYAGPASNDAIGITNLKSL   1019
Pikps-1     QPITINGTGFQMLESFRVDSRVPRIAFHEDAMPNLKLLEFKFYAGPASNDAIGITNLKSL   1019
Pi7-1       QPITINGTGFQMLESFRVDSRVPRIAFHEDAMPNLKLLEFKFYAGPASNDAIGITNLKSL   1019
Pik*-1      QPITINGTGFQMLESFRVDSRLPRIAFHEDAMPNLKLLEFKFYAGPASNDAIGITNLKSL   1020
Pike-1      QPITINGTGFQMLESFRVDSRVPRIAFHEDAMPNLKLLEFKFYAGPASNDAIGITNLKSL   1020
Pikg-1      QPITINGTGFQMLESFRVDSRVPRIAFHEDAMPNLKLLEFKFYAGPASNDAIGITNLKSL   1020
Pikm-1      QPITINGTGFQMLESFRVDSRVPRIAFHEDAMPNLKLLEFKFYAGPASNDAIGITNLKSL   1020
Pi1-5C      QPITINGTGFQMLESFRVDSRVPRIAFHEDAMPNLKLLEFKFYAGPASNDAIGITNLKSL   1020
Piks-1      QPITINGTGFQMLESFRVDSRVPRIAFHEDAMPNLKLLEFKFYAGPASNDAIGITNLKSL   1020
            *********************:**************************************
                                        LRR
Pikh-1      QKVVFRCSPWYKSDAPGISATIDVVKKEAEEHPNRPITLLINAGYKEISTESHGSSENIA   1079
Pikp-1      QKVVFRCSPWYKSDAPGISATIDVVKKEAEEHPNRPITLLINAGYKEISTESHGSSENIA   1079
Pikps-1     QKVVFRCSPWYKSDAPGISATIDVVKKEAEEHPNRPITLLINAGYKEISTESHGSSENIA   1079
Pi7-1       QKVVFRCSPWYKSDAPGISATIDVVKKEAEEHPNRPITLLINAGYKEISTESHGSSENIA   1079
Pik*-1      QKVVFRCSPWYKSDAPGISATIDVVKKEAEEHPNRPITLLINAGYKEISTESHGSSENIA   1080
Pike-1      QKVVFRCSPWYKSDAPGISATIDVVKKEAEEHPNRPITLLINAGYKEISTESHGSSENIA   1080
Pikg-1      QKVVFRCSPWYKSDAPGISATIDVVKKEAEEHPNRPITLLINAGYKEISTESHGSSENIA   1080
Pikm-1      QKVVFRCSPWYKSDAPGISATIDVVKKEAEEHPNRPITLLINAGYKEISTESHGSSENIA   1080
Pi1-5C      QKVVFRCSPWYKSDAPGISATIDVVKKEAEEHPNRPITLLINAGYKEISTESHGSSENIA   1080
Piks-1      QKVVFRCSPWYKSDAPGISATIDVVKKEAEEHPNRPITLLINAGYKEISTESHGSSENIA   1080
            ************************************************************
                                        LRR
Pikh-1      GSSGIDTEPAQAQHDNLPAVRDDYKGKGILLDGRCPTCGRATKIEEETQDRVADIEIQTE   1139
Pikp-1      GSSGIDTEPAQAQHDNLPAVRDDYKGKGILLDGRCPTCGRATKIEEETQDRVADIEIQTE   1139
```

```
Pikps-1      GSSGIDTEPAQAQHDNLPAVRDDYKGKGILLDGRCPTCGRATKIEEETQDRVADIEIQTE   1139
Pi7-1        GSSGIDTEPAQAQHDNLPAVRDDYKGKGILLDGRCPTCGRATKIEEETQDRVADIEIQTE   1139
Pik*-1       GSSGIDTEPAQAQHDNLPAVRDDYKGKGILLDGRCPTCGRATKIEEETQDRVADIEIQTE   1140
Pike-1       GSSGIDTEPAQAQHDNLPAVRDDYKGKGILLDGRCPTCGRATKIEEETQDRVADIEIQTE   1140
Pikg-1       GSSGIDTEPAQAQHDNLPAVRDDYKGKGILLDGRCPTCGRATKIEEETQDRVADIEIQTE   1140
Pikm-1       GSSGIDTEPAQAQHDNLPAVRDDYKGKGILLDGRCPTCGRATKIEEETQDRVADIEIQTE   1140
Pi1-5C       GSSGIDTEPAQAQHDNLPAVRDDYKGKGILLDGRCPTCGRATKIEEETQDRVADIEIQTE   1140
Piks-1       GSSGIDTEPAQAQHDNLPAVRDDYKGKGILLDGRCPTCGRATKIEEETQDRVADIEIQTE   1140
             ************************************************************
                                          LRR

Pikh-1       TTS 1142
Pikp-1       TTS 1142
Pikps-1      TTS 1142
Pi7-1        TTS 1142
Pik*-1       TTS 1143
Pike-1       TTS 1143
Pikg-1       TTS 1143
Pikm-1       TTS 1143
Pi1-5C       TTS 1143
Piks-1       TTS 1143
             ***
             LRR
```

Pik-2 alignment

```
Pikh-2      MELVVGASEATMKSLLGKLGNLLAQEYALISGIRGDIQYINDELASMQAFLRDLSNVPEG   60
Pikp-2      MELVVGASEATMKSLLGKLGNLLAQEYALISGIRGDIQYINDELASMQAFLRDLSNVPEG   60
Pikps-2     MELVVGASEATMKSLLGKLGNLLAQEYALISGIRGDIQYINDELASMQAFLRDLSNVPEG   60
Pi7-2       MELVVGASEATMKSLLGKLGNLLAQEYALISGIRGDIQYINDELASMQAFLRDLSNVPEG   60
Pike-2      MELVVGASEATMKSLLGKLGNLLAQEYALISGIRGDIQYINDELASMQAFLRDLSNVPEG   60
Pikg-2      MELVVGASEATMKSLLGKLGNLLAQEYALISGIRGDIQYINDELASMQAFLRDLSNVPEG   60
Pik*-2      MELVVGASEATMKSLLGKLGNLLAQEYALISGIRGDIQYINDELASMQAFLRDLSNVPEG   60
Pikm-2      MELVVGASEATMKSLLGKLGNLLAQEYALISGIRGDIQYINDELASMQAFLRDLSNVPEG   60
Piks-2      MELVVGASEATMKSLLGKLGNLLAQEYALISGIRGDIQYINDELASMQAFLRDLSNVPEG   60
Pi1-6C      MELVVGASEATMKSLLGKLGNLLAQEYALISGIRGDIQYINDELASMQAFLRDLSNVPEG   60
            ************************************************************
                                        CC
Pikh-2      HSHGHRMKDWMKQIRDIAYDVEDCIDDFAHRLPQDSISDAKWSFLLTKIYELWTWWPRRV   120
Pikp-2      HSHGHRMKDWMKQIRDIAYDVEDCIDDFAHRLPQDSISDAKWSFLLTKIYELWTWWPRRV   120
Pikps-2     HSHGHRMKDWMKQIRDIAYDVEDCIDDFAHRLPQDSISDAKWSFLLTKIYELWTWWPRRV   120
Pi7-2       HSHGHRMKDWMKQIRDIAYDVEDCIDDFAHRLPQDSISDAKWSFLLTKIYELWTWWPRRV   120
Pike-2      HSHGHRMKDWMKQIRDIAYDVEDCIDDFAHRLPQDSISDAKWSFLLTKIYELWTWWPRRV   120
Pikg-2      HSHGHRMKDWMKQIRDIAYDVEDCIDDFAHRLPQDSISDAKWSFLLTKIYELWTWWPRRV   120
Pik*-2      HSHGHRMKDWMKQIRDIAYDVEDCIDDFAHRLPQDSISDAKWSFLLTKIYELWTWWPRRV   120
Pikm-2      HSHGHRMKDWMKQIRDIAYDVEDCIDDFAHRLPQDSISDAKWSFLLTKIYELWTWWPRRV   120
Piks-2      HSHGHRMKDWMKQIRDIAYDVEDCIDDFAHRLPQDSISDAKWSFLLTKIYELWTWWPRRV   120
Pi1-6C      HSHGHRMKDWMKQIRDIAYDVEDCIDDFAHRLPQDSISDAKWSFLLTKIYELWTWWPRRV   120
            ************************************************************
                                        CC
Pikh-2      IASNIAQLKVRAQQIADRRSRYGVNNPEHLDSSSSARTRAVNYEIAEYQVTSPQIIGIKE   180
Pikp-2      IASNIAQLKVRAQQIADRRSRYGVNNPEHLDSSSSARTRAVNYEIAEYQVTSPQIIGIKE   180
Pikps-2     IASNIAQLKVRAQQIADRRSRYGVNNPEHLDSSSSARTRAVNYEIAEYQVTSPQIIGIKE   180
Pi7-2       IASNIAQLKVRAQQIADRRSRYGVNNPEHLDSSSSARTRAVNYEIAEYQVTSPQIIGIKE   180
Pike-2      IASNIAQLKVRAQQIADRRSRYGVNNPEHLDSSSSARTRAVNYEIAEYQVTSPQIIGIKE   180
Pikg-2      IASNIAQLKVRAQQIADRRSRYGVNNPEHLDSSSSARTRAVNYEIAEYQVTSPQIIGIKE   180
Pik*-2      IASNIAQLKVRAQQIADRRSRYGVNNPEHLDSSSSARTRAVNYEIAEYQVTSPQIIGIKE   180
Pikm-2      IASNIAQLKVRAQQIADRRSRYGVNNPEHLDSSSSARTRAVNYEIAEYQVTSPQIIGIKE   180
Piks-2      IASNIAQLKVRAQQIADRRSRYGVNNPEHLDSSSSARTRAVNYEIAEYQVTSPQIIGIKE   180
Pi1-6C      IASNIAQLKVRAQQIADRRSRYGVNNPEHLDSSSSARTRAVNYEIAEYQVTSPQIIGIKE   180
            ************************************************************
                                        CC
Pikh-2      PVGMKTVMEELEVWLTNPQAENGQAVLSIVGFGGVGKTTIATALYRKVSDKFQCRASVAV   240
Pikp-2      PVGMKTVMEELEVWLTNPQAENGQAVLSIVGFGGVGKTTIATALYRKVSDKFQCRASVAV   240
Pikps-2     PVGMKTVMEELEVWLTNPQAENGQAVLSIVGFGGVGKTTIATALYRKVSDKFQCRASVAV   240
Pi7-2       PVGMKTVMEELEVWLTNPQAENGQAVLSIVGFGGVGKTTIATALYRKVSDKFQCRASVAV   240
Pike-2      PVGMKTVMEELEVWLTNPQAENGQAVLSIVGFGGVGKTTIATALYRKVSDKFQCRASVAV   240
Pikg-2      PVGMKTVMEELEVWLTNPQAENGQAVLSIVGFGGVGKTTIATALYRKVSDKFQCRASVAV   240
Pik*-2      PVGMKTVMEELEVWLTNPQAENGQAVLSIVGFGGVGKTTIATALYRKVSEKFQCRASVAV   240
Pikm-2      PVGMKTVMEELEVWLTNPQAENGQAVLSIVGFGGVGKTTIATALYRKVSEKFQCRASVAV   240
Piks-2      PVGMKTVMEELEVWLTNPQAENGQAVLSIVGFGGVGKTTIATALYRKVSEKFQCRASVAV   240
Pi1-6C      PVGMKTVMEELEVWLTNPQAENGQAVLSIVGFGGVGKTTIATALYRKVSEKFQCRASVAV   240
            *************************************************:**********
             CC 185                            NB-ARC
Pikh-2      SQNYDQGKVLNSILSQVSNQEQGSSTTISEKKNLTSGAKSMLKTALSLLRGNCICQPEND   300
Pikp-2      SQNYDQGKVLNSILSQVSNQEQGSSTTISEKKNLTSGAKSMLKTALSLLRGNCICQPEND   300
Pikps-2     SQNYDQGKVLNSILSQVSNQEQGSSTTISEKKNLTSGAKSMLKTALSLLRGNCICQPEND   300
Pi7-2       SQNYDQGKVLNSILSQVSNQEQGSSTTISEKKNLTSGAKSMLKTALSLLRGNCICQPEND   300
Pike-2      SQNYDQGKVLNSILSQVSNQEQGSSTTISEKKNLTSGAKSMLKTALSLLRGNCICQPEND   300
Pikg-2      SQNYDQGKVLNSILSQVSNQEQGSSTTISEKKNLTSGAKSMLKTALSLLRGNCICQPEND   300
```

```
Pik*-2      SQNYDQGKVLNSILSQVSNQEQGSSTTISEKKNLTSGAKSMLKTALSLLRGNCICQPEND   300
Pikm-2      SQNYDQGKVLNSILSQVSNQEQGSSTTISEKKNLTSGAKSMLKTALSLLRGNCICQPEND   300
Piks-2      SQNYDQGKVLNSILSQVSNQEQGSSTTISEKKNLTSGAKSMLKTALSLLRGNCICQPEND   300
Pi1-6C      SQNYDQGKVLNSILSQVSNQEQGSSTTISEKKNLTSGAKSMLKTALSLLRGNCICQPEND   300
            ************************************************************
                                    NB-ARC
Pikh-2      GNPDNTPIRLQETTDDDQNPRKLEQLLAEKSYILLIDDIWSAETWESIRSILPKNNKGGR   360
Pikp-2      GNPDNTPIRLQETTDDDQNPRKLEQLLAEKSYILLIDDIWSAETWESIRSILPKNNKGGR   360
Pikps-2     GNPDNTPIRLQETTDDDQNPRKLEQLLAEKSYILLIDDIWSAETWESIRSILPKNNKGGR   360
Pi7-2       GNPDNTPIRLQETTDDDQNPRKLEQLLAEKSYILLIDDIWSAETWESIRSILPKNNKGGR   360
Pike-2      GNPDNTPIRLQETTDDDQNPRKLEQLLAEKSYILLIDDIWSAETWESIRSILPKNNKGGR   360
Pikg-2      GNPDNTPIRLQETTDDDQNPRKLEQLLAEKSYILLIDDIWSAETWESIRSILPKNNKGGR   360
Pik*-2      GNPDNTPIRLQETTDDDQNPRKLEQLLAEKSYILLIDDIWSAETWESIRSILPKNNKGGR   360
Pikm-2      GNPDNTPIRLQETTDDDQNPRKLEQLLAEKSYILLIDDIWSAETWESIRSILPKNNKGGR   360
Piks-2      GNPDNTPIRLQETTDDDQNPRKLEQLLAEKSYILLIDDIWSAETWESIRSILPKNNKGGR   360
Pi1-6C      GNPDNTPIRLQETTDDDQNPRKLEQLLAEKSYILLIDDIWSAETWESIRSILPKNNKGGR   360
            ************************************************************
                                    NB-ARC
Pikh-2      IIVTTRFQAVGSTCSPLETDRLHTVDFLTDDESQNLFNTSICESKIRKDSNKVDEQVPEE   420
Pikp-2      IIVTTRFQAVGSTCSPLETDRLHTVDFLTDDESQNLFNTSICESKIRKDSNKVDEQVPEE   420
Pikps-2     IIVTTRFQAVGSTCSPLETDRLHTVDFLTDDESQNLFNTSICESKIRKDSNKVDEQVPEE   420
Pi7-2       IIVTTRFQAVGSTCSPLETDRLHTVDFLTDDESQNLFNTSICESKIRKDSNKVDEQVPEE   420
Pike-2      IIVTTRFQAVGSTCSPLETDRLHTVDFLTDDESQNLFNTSICESKIRKDSNKVDEQVPEE   420
Pikg-2      IIVTTRFQAVGSTCSPLETDRLHTVDFLTDDESQNLFNTSICESKIRKDSNKVDEQVPEE   420
Pik*-2      IIVTTRFQAVGSTCSPLETDRLHTVDFLTDDESQNLFNTSICESKIRKDSNKVDEQVPEE   420
Pikm-2      IIVTTRFQAVGSTCSPLETDRLHTVDFLTDDESQNLFNTSICESKIRKDSNKVDEQVPEE   420
Piks-2      IIVTTRFQAVGSTCSPLETDRLHTVDFLTDDESQNLFNTSICESKIRKDSNKVDEQVPEE   420
Pi1-6C      IIVTTRFQAVGSTCSPLETDRLHTVDFLTDDESQNLFNTSICESKIRKDSNKVDEQVPEE   420
            ************************************************************
                                    NB-ARC
Pikh-2      IWKICGGLPLAIVTMAGLVACNPRKACCDWSKLCKSLFPEQETPLTLDGVTRILDCCYND   480
Pikp-2      IWKICGGLPLAIVTMAGLVACNPRKACCDWSKLCKSLFPEQETPLTLDGVTRILDCCYND   480
Pikps-2     IWKICGGLPLAIVTMAGLVACNPRKACCDWSKLCKSLFPEQETPLTLDGVTRILDCCYND   480
Pi7-2       IWKICGGLPLAIVTMAGLVACNPRKACCDWSKLCKSLFPEQETPLTLDGVTRILDCCYND   480
Pike-2      IWKICGGLPLAIVTMAGLVACNPRKACCDWSKLCKSLFPEQETPLTLDGVTRILDCCYND   480
Pikg-2      IWKICGGLPLAIVTMAGLVACNPRKACCDWSKLCKSLFPEQETPLTLDGVTRILDCCYND   480
Pik*-2      IWKICGGLPLAIVSMAGLVACNPRKACCDWSKLCKSLFPEQETPLTLDGVTRILDCCYND   480
Pikm-2      IWKICGGLPLAIVSMAGLVACNPRKACCDWSKLCKSLFPEQETPLTLDGVTRILDCCYND   480
Piks-2      IWKICGGLPLAIVSMAGLVACNPRKACCDWSKLCKSLFPEQETPLTLDGVTRILDCCYND   480
Pi1-6C      IWKICGGLPLAIVSMAGLVACNPRKACCDWSKLCKSLFPEQETPLTLDGVTRILDCCYND   480
            *************:**********************************************
                                    NB-ARC
Pikh-2      LPADLKTCLLYLSIFPKGWKISRKRLSRRWIAEGFANEKQGLTQERVAEAYFNQLTRRNL   540
Pikp-2      LPADLKTCLLYLSIFPKGWKISRKRLSRRWIAEGFANEKQGLTQERVAEAYFNQLTRRNL   540
Pikps-2     LPADLKTCLLYLSIFPKGWKISRKRLSRRWIAEGFANEKQGLTQERVAEAYFNQLTRRNL   540
Pi7-2       LPADLKTCLLYLSIFPKGWKISRKRLSRRWIAEGFANEKQGLTQERVAEAYFNQLTRRNL   540
Pike-2      LPADLKTCLLYLSIFPKGWKISRKRLSRRWIAEGFANEKQGLTQERVAEAYFNQLTRRNL   540
Pikg-2      LPADLKTCLLYLSIFPKGWKISRKRLSRRWIAEGFANEKQGLTQERVAEAYFNQLTRRNL   540
Pik*-2      LPADLKTCLLYLSIFPKGWKISRKRLSRRWIAEGFANEKQGLTQERVAEAYFNQLTRRNL   540
Pikm-2      LPADLKTCLLYLSIFPKGWKISRKRLSRRWIAEGFANEKQGLTQERVAEAYFNQLTRRNL   540
Piks-2      LPADLKTCLLYLSIFPKGWKISRKRLSRRWIAEGFANEKQGLTQERVAEAYFNQLTRRNL   540
Pi1-6C      LPADLKTCLLYLSIFPKGWKISRKRLSRRWIAEGFANEKQGLTQERVAEAYFNQLTRRNL   540
            ************************************************************
                                    NB-ARC
Pikh-2      VRPMEHGSNGKVKTFQVHDMVLEYIMSKSIEENFITVVGGHWQMTAPSNKVRRLSMQSSG   600
Pikp-2      VRPMEHGSNGKVKTFQVHDMVLEYIMSKSIEENFITVVGGHWQMTAPSNKVRRLSMQSSG   600
```

```
Pikps-2      VRPMEHGSNGKVKTFQVHDMVLEYIMSKSIEENFITVVGGHWQMTAPSNKVRRLSMQSSG   600
Pi7-2        VRPMEHGSNGKVKTFQVHDMVLEYIMSKSIEENFITVVGGHWQMTAPSNKVRRLSMQSSG   600
Pike-2       VRPMEHGSNGKVKTFQVHDMVLEYIMSKSIEENFITVVGGHWQMTAPSNKVRRLSMQSSG   600
Pikg-2       VRPMEHGSNGKVKTFQVHDMVLEYIMSKSIEENFITVVGGHWQMTAPSNKVRRLSMQSSG   600
Pik*-2       VRPMEHGSNGKVKTFQVHDMVLEYIMSKSIEENFITVVGGHWQMTAPSNKVRRLSMQSSG   600
Pikm-2       VRPMEHGSNGKVKTFQVHDMVLEYIMSKSIEENFITVVGGHWQMTAPSNKVRRLSMQSSG   600
Piks-2       VRPMEHGSNGKVKTFQVHDMVLEYIMSKSIEENFITVVGGHWQMTAPSNKVRRLSMQSSG   600
Pi1-6C       VRPMEHGSNGKVKTFQVHDMVLEYIMSKSIEENFITVVGGHWQMTAPSNKVRRLSMQSSG   600
             ************************************************************
                                          NB-ARC
Pikh-2       SNRGSSTKGLNLAQVRSLTVFGNLNHMPFHSFNYGIIQVLDLEDWKGLKERHMTEICQML   660
Pikp-2       SNRGSSTKGLNLAQVRSLTVFGNLNHMPFHSFNYGIIQVLDLEDWKGLKERHMTEICQML   660
Pikps-2      SNRGSSTKGLNLAQVRSLTVFGNLNHMPFHSFNYGIIQVLDLEDWKGLKERHMTEICQML   660
Pi7-2        SNRGSSTKGLNLAQVRSLTVFGNLNHMPFHSFNYGIIQVLDLEDWKGLKERHMTEICQML   660
Pike-2       SNRGSSTKGLNLAQVRSLTVFGNLNHVPFHSFNYGIIQVLDLEDWKGLKERHMTEICQML   660
Pikg-2       SNRGSSTKGLNLAQVRSLTVFGNLNHVPFHSFNYGIIQVLDLEDWKGLKERHMTEICQML   660
Pik*-2       SNRGSSTKGLNLAQVRSLTVFGNLNHVPFHSFNYGIIQVLDLEDWKGLKERHMTEICQML   660
Pikm-2       SNRGSSTKGLNLAQVRSLTVFGNLNHVPFHSFNYGIIQVLDLEDWKGLKERHMTEICQML   660
Piks-2       SNRGSSTKGLNLAQVRSLTVFGNLNHVPFHSFNYGIIQVLDLEDWKGLKERHMTEICQML   660
Pi1-6C       SNRGSSTKGLNLAQVRSLTVFGNLNHVPFHSFNYGIIQVLDLEDWKGLKERHMTEICQML   660
             **************************:*********************************
               NB-ARC  611                      LRR
Pikh-2       LLKYLSIRRTEISKIPSKIQKLEYLETLDIRETYVRDLPKSIVQLKRIISILGGNKNTRK   720
Pikp-2       LLKYLSIRRTEISKIPSKIQKLEYLETLDIRETYVRDLPKSIVQLKRIISILGGNKNTRK   720
Pikps-2      LLKYLSIRRTEISKIPSKIQKLEYLETLDIRETYVRDLPKSIVQLKRIISILGGNKNTRK   720
Pi7-2        LLKYLSIRRTEISKIPSKIQKLEYLETLDIRETYVRDLPKSIVQLKRIISILGGNKNTRK   720
Pike-2       LLKYLSIRRTEISKIPSKIQKLEYLETLDIRETYVRDLPKSIVQLKRIISILGGNKNTRK   720
Pikg-2       LLKYLSIRRTEISKIPSKIQKLEYLETLDIRETYVRDLPKSIVQLKRIISILGGNKNTRK   720
Pik*-2       LLKYLSIRRTEISKIPSKIQKLEYLETLDIRETYVRDLPKSIVQLKRIISILGGNKNTRK   720
Pikm-2       LLKYLSIRRTEISKIPSKIQKLEYLETLDIRETYVRDLPKSIVQLKRIISILGGNKNTRK   720
Piks-2       LLKYLSIRRTEISKIPSKIQKLEYLETLDIRETYVRDLPKSIVQLKRIISILGGNKNTRK   720
Pi1-6C       LLKYLSIRRTEISKIPSKIQKLEYLETLDIRETYVRDLPKSIVQLKRIISILGGNKNTRK   720
             ************************************************************
                                           LRR
Pikh-2       GLRLPQEKSKKPIKNPSPQGKTKEPAKKGFLSQEKGKGAMKALRVLSGIEIVEESSEVAA   780
Pikp-2       GLRLPQEKSKKPIKNPSPQGKTKEPAKKGFLSQEKGKGAMKALRVLSGIEIVEESSEVAA   780
Pikps-2      GLRLPQEKSKKPIKNPSPQGKTKEPAKKGFLSQEKGKGAMKALRVLSGIEIVEESSEVAA   780
Pi7-2        GLRLPQEKSKKPIKNPSPQGKTKEPAKKGFLSQEKGKGAMKALRVLSGIEIVEESSEVAA   780
Pike-2       GLRLPQEKSKKPIKNPSPQGKTKEPAKKGFLSQEKGKGAMKALRVLSGIEIVEESSEVAA   780
Pikg-2       GLRLPQEKSKKPIKNPSPQGKTKEPAKKGFLSQEKGKGAMKALRVLSGIEIVEESSEVAA   780
Pik*-2       GLRLPQEKSKKPIKNPSPQGKTKEPAKKGFLSQEKGKGAMKALRVLSGIEIVEESSEVAA   780
Pikm-2       GLRLPQEKSKKPIKNPSPQGKTKEPAKKGFLSQEKGKGAMKALRVLSGIEIVEESSEVAA   780
Piks-2       GLRLPQEKSKKPIKNPSPQGKTKEPAKKGFLSQEKGKGAMKALRVLSGIEIVEESSEVAA   780
Pi1-6C       GLRLPQEKSKKPIKNPSPQGKTKEPAKKGFLSQVKGKGAMKALRVLSGIEIVEESSEVAA   780
             ********************************* **************************
                                           LRR
Pikh-2       GLHQLTGLRKLAIYKLNITKGGDTFKQLQSSIEYLGSCGLQTLAINDENSEFINSLGDMP   840
Pikp-2       GLHQLTGLRKLAIYKLNITKGGDTFKQLQSSIEYLGSCGLQTLAINDENSEFINSLGDMP   840
Pikps-2      GLHQLTGLRKLAIYKLNITKGGDTFKQLQSSIEYLGSCGLQTLAINDENSEFINSLGDMP   840
Pi7-2        GLHQLTGLRKLAIYKLNITKGGDTFKQLQSSIEYLGSCGLQTLAINDENSEFINSLGDMP   840
Pike-2       GLHQLTGLRKLAIYKLNITKGGDTFKQLQSSIEYLGSCGLQTLAINDENSEFINSLGDMP   840
Pikg-2       GLHQLTGLRKLAIYKLNITKGGDTFKQLQSSIEYLGSCGLQTLAINDENSEFINSLGDMP   840
Pik*-2       GLHQLTGLRKLAIYKLNITKGGDTFKQLQSSIEYLGSCGLQTLAINDENSEFINSLGDMP   840
Pikm-2       GLHQLTGLRKLAIYKLNITKGGDTFKQLQSSIEYLGSCGLQTLAINDENSEFINSLGDMP   840
Piks-2       GLHQLTGLRKLAIYKLNITKGGDTFKQLQSSIEYLGSCGLQTLAINDENSEFINSLGDMP   840
```

```
Pi1-6C       GLHQLTGLRKLAIYKLNITKGGDTFKQLQSSIEYLGSCGLQTLAINDENSEFINSLGDMP   840
             ************************************************************
                                         LRR
Pikh-2       APPRYLVALELSGKLEKLPKWITSITTLNKLTISVTVLRTETLEILHILPSLFSLTFAFS   900
Pikp-2       APPRYLVALELSGKLEKLPKWITSITTLNKLTISVTVLRTETLEILHILPSLFSLTFAFS   900
Pikps-2      APPRYLVALELSGKLEKLPKWITSITTLNKLTISVTVLRTETLEILHILPSLFSLTFAFS   900
Pi7-2        APPRYLVALELSGKLEKLPKWITSITTLNKLTISVTVLRTETLEILHILPSLFSLTFAFS   900
Pike-2       APPRYLVALELSGKLEKLPKWITSITTLNKLTISVTVLRTETLEILHILPSLFSLTFAFS   900
Pikg-2       APPRYLVALELSGKLEKLPKWITSITTLNKLTISVTVLRTETLEILHILPSLFSLTFAFS   900
Pik*-2       APPRYLVALELSGKLEKLPKWITSITTLNKLTISVTVLRTETLEILHILPSLFSLTFAFS   900
Pikm-2       APPRYLVALELSGKLEKLPKWITSITTLNKLTISVTVLRTETLEILHILPSLFSLTFAFS   900
Piks-2       APPRYLVALELSGKLEKLPKWITSITTLNKLTISVTVLRTETLEILHILPSLFSLTFAFS   900
Pi1-6C       APPRYLVALELSGKLEKLPKWITSITTLNKLTISVTVLRTETLEILHILPSLFSLTFAFS   900
             ************************************************************
                                         LRR
Pikh-2       LSAAKQDQDIIKDILENNKLDSDGEIVIPAEGFKSLKLLRFFAPLVPKLSFLDKNAMPAL   960
Pikp-2       LSAAKQDQDIIKDILENNKLDSDGEIVIPAEGFKSLKLLRFFAPLVPKLSFLDKNAMPAL   960
Pikps-2      LSAAKQDQDIIKDILENNKLDSDGEIVIPAEGFKSLKLLRFFAPLVPKLSFLDKNAMPAL   960
Pi7-2        LSAAKQDQDIIKDILENNKLDSDGEIVIPAEGFKSLKLLRFFAPLVPKLSFLDKNAMPAL   960
Pike-2       LSAAKQDQDIIKDILENNKLDSDGEIVIPAEGFKSLKLLRFFAPLVPKLSFLDKNAMPAL   960
Pikg-2       LSAAKQDQDIIKDILENNKLDSDGEIVIPAEGFKSLKLLRFFAPLVPKLSFLDKNAMPAL   960
Pik*-2       LSAAKQDQDIIKDILENNKLDSDGEIVIPAEGFKSLKLLRFFAPLVPKLSFLDKNAMPAL   960
Pikm-2       LSAAKQDQDIIKDILENNKLDSDGEIVIPAEGFKSLKLLRFFAPLVPKLSFLDKNAMPAL   960
Piks-2       LSAAKQDQDIIKDILENNKLDSDGEIVIPAEGFKSLKLLRFFAPLVPKLSFLDKNAMPAL   960
Pi1-6C       LSAAKQDQDIIKDILENNKLDSDGEIVIPAEGFKSLKLLRFFAPLVPKLSFLDKNAMPAL   960
             ************************************************************
                                         LRR
Pikh-2       EIIEMRFKDFEGLFGIEILENLREVHLKVSDGAEAITKFLVNDLKVNTEKPKVFVDGIVT   1020
Pikp-2       EIIEMRFKDFEGLFGIEILENLREVHLKVSDGAEAITKFLVNDLKVNTEKPKVFVDGIVT   1020
Pikps-2      EIIEMRFKDFEGLFGIEILENLREVHLKVSDGAEAITKFLVNDLKVNTEKPKVFVDGIVT   1020
Pi7-2        EIIEMRFKDFEGLFGIEILENLREVHLKVSDGAEAITKFLVNDLKVNTEKPKVFVDGIVT   1020
Pike-2       EIIEMRFKDFEGLFGIEILENLREVHLKVSDGAEAITKFLVNDLKDNTEKPKVFVDGIVT   1020
Pikg-2       EIIEMRFKDFEGLFGIEILENLREVHLKVSDGAEAITKFLVNDLKDNTEKPKVFVDGIVT   1020
Pik*-2       EIIEMRFKDFEGLFGIEILENLREVHLKVSDGAEAITKFLVNDLKVNTEKPKVFVDGIVT   1020
Pikm-2       EIIEMRFKDFEGLFGIEILENLREVHLKVSDGAEAITKFLVNDLKVNTEKPKVFVDGIVT   1020
Piks-2       EIIEMRFKDFEGLFGIEILENLREVHLKVSDGAEAITKFLVNDLKVNTEKPKVFVDGIVT   1020
Pi1-6C       EIIEMRFKDFEGLFGIEILENLREVHLKVSDGAEAITKFLVNDLKVNTEKPKVFVDGIVT   1020
             ********************************************* **************
                                         LRR
Pikh-2       A-   1021
Pikp-2       A-   1021
Pikps-2      A-   1021
Pi7-2        A-   1021
Pike-2       A-   1021
Pikg-2       A-   1021
Pik*-2       A-   1021
Pikm-2       A-   1021
Piks-2       A-   1021
Pi1-6C       A-   1021
             *
            LRR
```

References

Bentham AR, De la Concepcion JC, Benjumea JV, Kourelis J, Jones S, Mendel M, Stubbs J, Stevenson CEM, Maidment JHR, Youles M, et al. 2023. Allelic compatibility in plant immune receptors facilitates engineering of new effector recognition specificities. *Plant Cell* **35**(10): 3809-3827.

De la Concepcion JC, Franceschetti M, Maqbool A, Saitoh H, Terauchi R, Kamoun S, Banfield MJ. 2018. Polymorphic residues in rice NLRs expand binding and response to effectors of the blast pathogen. *Nat Plants* **4**(8): 576-585.

Maidment JHR. 2020. *Using a rice blast effector target to engineer NLR immune receptors with novel recognition specificities.* Thesis (Doctoral), University of East Anglia.
